# Measurement of the $t\overline{t}$ production cross-section using $e\mu$ events with $b$-tagged jets in $pp$ collisions at $\sqrt{s}=7$ and 8 TeV with the ATLAS detector

**DOI:** 10.1140/epjc/s10052-014-3109-7

**Published:** 2014-10-29

**Authors:** G. Aad, B. Abbott, J. Abdallah, S. Abdel Khalek, O. Abdinov, R. Aben, B. Abi, M. Abolins, O. S. AbouZeid, H. Abramowicz, H. Abreu, R. Abreu, Y. Abulaiti, B. S. Acharya, L. Adamczyk, D. L. Adams, J. Adelman, S. Adomeit, T. Adye, T. Agatonovic-Jovin, J. A. Aguilar-Saavedra, M. Agustoni, S. P. Ahlen, F. Ahmadov, G. Aielli, H. Akerstedt, T. P. A. Åkesson, G. Akimoto, A. V. Akimov, G. L. Alberghi, J. Albert, S. Albrand, M. J. Alconada Verzini, M. Aleksa, I. N. Aleksandrov, C. Alexa, G. Alexander, G. Alexandre, T. Alexopoulos, M. Alhroob, G. Alimonti, L. Alio, J. Alison, B. M. M. Allbrooke, L. J. Allison, P. P. Allport, J. Almond, A. Aloisio, A. Alonso, F. Alonso, C. Alpigiani, A. Altheimer, B. Alvarez Gonzalez, M. G. Alviggi, K. Amako, Y. Amaral Coutinho, C. Amelung, D. Amidei, S. P. Amor Dos Santos, A. Amorim, S. Amoroso, N. Amram, G. Amundsen, C. Anastopoulos, L. S. Ancu, N. Andari, T. Andeen, C. F. Anders, G. Anders, K. J. Anderson, A. Andreazza, V. Andrei, X. S. Anduaga, S. Angelidakis, I. Angelozzi, P. Anger, A. Angerami, F. Anghinolfi, A. V. Anisenkov, N. Anjos, A. Annovi, A. Antonaki, M. Antonelli, A. Antonov, J. Antos, F. Anulli, M. Aoki, L. Aperio Bella, R. Apolle, G. Arabidze, I. Aracena, Y. Arai, J. P. Araque, A. T. H. Arce, J-F. Arguin, S. Argyropoulos, M. Arik, A. J. Armbruster, O. Arnaez, V. Arnal, H. Arnold, M. Arratia, O. Arslan, A. Artamonov, G. Artoni, S. Asai, N. Asbah, A. Ashkenazi, B. Åsman, L. Asquith, K. Assamagan, R. Astalos, M. Atkinson, N. B. Atlay, B. Auerbach, K. Augsten, M. Aurousseau, G. Avolio, G. Azuelos, Y. Azuma, M. A. Baak, A. Baas, C. Bacci, H. Bachacou, K. Bachas, M. Backes, M. Backhaus, J. Backus Mayes, E. Badescu, P. Bagiacchi, P. Bagnaia, Y. Bai, T. Bain, J. T. Baines, O. K. Baker, P. Balek, F. Balli, E. Banas, Sw. Banerjee, A. A. E. Bannoura, V. Bansal, H. S. Bansil, L. Barak, S. P. Baranov, E. L. Barberio, D. Barberis, M. Barbero, T. Barillari, M. Barisonzi, T. Barklow, N. Barlow, B. M. Barnett, R. M. Barnett, Z. Barnovska, A. Baroncelli, G. Barone, A. J. Barr, F. Barreiro, J. Barreiro Guimarães da Costa, R. Bartoldus, A. E. Barton, P. Bartos, V. Bartsch, A. Bassalat, A. Basye, R. L. Bates, J. R. Batley, M. Battaglia, M. Battistin, F. Bauer, H. S. Bawa, M. D. Beattie, T. Beau, P. H. Beauchemin, R. Beccherle, P. Bechtle, H. P. Beck, K. Becker, S. Becker, M. Beckingham, C. Becot, A. J. Beddall, A. Beddall, S. Bedikian, V. A. Bednyakov, C. P. Bee, L. J. Beemster, T. A. Beermann, M. Begel, K. Behr, C. Belanger-Champagne, P. J. Bell, W. H. Bell, G. Bella, L. Bellagamba, A. Bellerive, M. Bellomo, K. Belotskiy, O. Beltramello, O. Benary, D. Benchekroun, K. Bendtz, N. Benekos, Y. Benhammou, E. Benhar Noccioli, J. A. Benitez Garcia, D. P. Benjamin, J. R. Bensinger, K. Benslama, S. Bentvelsen, D. Berge, E. Bergeaas Kuutmann, N. Berger, F. Berghaus, J. Beringer, C. Bernard, P. Bernat, C. Bernius, F. U. Bernlochner, T. Berry, P. Berta, C. Bertella, G. Bertoli, F. Bertolucci, C. Bertsche, D. Bertsche, M. I. Besana, G. J. Besjes, O. Bessidskaia, M. F. Bessner, N. Besson, C. Betancourt, S. Bethke, W. Bhimji, R. M. Bianchi, L. Bianchini, M. Bianco, O. Biebel, S. P. Bieniek, K. Bierwagen, J. Biesiada, M. Biglietti, J. Bilbao De Mendizabal, H. Bilokon, M. Bindi, S. Binet, A. Bingul, C. Bini, C. W. Black, J. E. Black, K. M. Black, D. Blackburn, R. E. Blair, J.-B. Blanchard, T. Blazek, I. Bloch, C. Blocker, W. Blum, U. Blumenschein, G. J. Bobbink, V. S. Bobrovnikov, S. S. Bocchetta, A. Bocci, C. Bock, C. R. Boddy, M. Boehler, T. T. Boek, J. A. Bogaerts, A. G. Bogdanchikov, A. Bogouch, C. Bohm, J. Bohm, V. Boisvert, T. Bold, V. Boldea, A. S. Boldyrev, M. Bomben, M. Bona, M. Boonekamp, A. Borisov, G. Borissov, M. Borri, S. Borroni, J. Bortfeldt, V. Bortolotto, K. Bos, D. Boscherini, M. Bosman, H. Boterenbrood, J. Boudreau, J. Bouffard, E. V. Bouhova-Thacker, D. Boumediene, C. Bourdarios, N. Bousson, S. Boutouil, A. Boveia, J. Boyd, I. R. Boyko, J. Bracinik, A. Brandt, G. Brandt, O. Brandt, U. Bratzler, B. Brau, J. E. Brau, H. M. Braun, S. F. Brazzale, B. Brelier, K. Brendlinger, A. J. Brennan, R. Brenner, S. Bressler, K. Bristow, T. M. Bristow, D. Britton, F. M. Brochu, I. Brock, R. Brock, C. Bromberg, J. Bronner, G. Brooijmans, T. Brooks, W. K. Brooks, J. Brosamer, E. Brost, J. Brown, P. A. Bruckman de Renstrom, D. Bruncko, R. Bruneliere, S. Brunet, A. Bruni, G. Bruni, M. Bruschi, L. Bryngemark, T. Buanes, Q. Buat, F. Bucci, P. Buchholz, R. M. Buckingham, A. G. Buckley, S. I. Buda, I. A. Budagov, F. Buehrer, L. Bugge, M. K. Bugge, O. Bulekov, A. C. Bundock, H. Burckhart, S. Burdin, B. Burghgrave, S. Burke, I. Burmeister, E. Busato, D. Büscher, V. Büscher, P. Bussey, C. P. Buszello, B. Butler, J. M. Butler, A. I. Butt, C. M. Buttar, J. M. Butterworth, P. Butti, W. Buttinger, A. Buzatu, M. Byszewski, S. Cabrera Urbán, D. Caforio, O. Cakir, P. Calafiura, A. Calandri, G. Calderini, P. Calfayan, R. Calkins, L. P. Caloba, D. Calvet, S. Calvet, R. Camacho Toro, S. Camarda, D. Cameron, L. M. Caminada, R. Caminal Armadans, S. Campana, M. Campanelli, A. Campoverde, V. Canale, A. Canepa, M. Cano Bret, J. Cantero, R. Cantrill, T. Cao, M. D. M. Capeans Garrido, I. Caprini, M. Caprini, M. Capua, R. Caputo, R. Cardarelli, T. Carli, G. Carlino, L. Carminati, S. Caron, E. Carquin, G. D. Carrillo-Montoya, J. R. Carter, J. Carvalho, D. Casadei, M. P. Casado, M. Casolino, E. Castaneda-Miranda, A. Castelli, V. Castillo Gimenez, N. F. Castro, P. Catastini, A. Catinaccio, J. R. Catmore, A. Cattai, G. Cattani, S. Caughron, V. Cavaliere, D. Cavalli, M. Cavalli-Sforza, V. Cavasinni, F. Ceradini, B. Cerio, K. Cerny, A. S. Cerqueira, A. Cerri, L. Cerrito, F. Cerutti, M. Cerv, A. Cervelli, S. A. Cetin, A. Chafaq, D. Chakraborty, I. Chalupkova, P. Chang, B. Chapleau, J. D. Chapman, D. Charfeddine, D. G. Charlton, C. C. Chau, C. A. Chavez Barajas, S. Cheatham, A. Chegwidden, S. Chekanov, S. V. Chekulaev, G. A. Chelkov, M. A. Chelstowska, C. Chen, H. Chen, K. Chen, L. Chen, S. Chen, X. Chen, Y. Chen, Y. Chen, H. C. Cheng, Y. Cheng, A. Cheplakov, R. Cherkaoui El Moursli, V. Chernyatin, E. Cheu, L. Chevalier, V. Chiarella, G. Chiefari, J. T. Childers, A. Chilingarov, G. Chiodini, A. S. Chisholm, R. T. Chislett, A. Chitan, M. V. Chizhov, S. Chouridou, B. K. B. Chow, D. Chromek-Burckhart, M. L. Chu, J. Chudoba, J. J. Chwastowski, L. Chytka, G. Ciapetti, A. K. Ciftci, R. Ciftci, D. Cinca, V. Cindro, A. Ciocio, P. Cirkovic, Z. H. Citron, M. Citterio, M. Ciubancan, A. Clark, P. J. Clark, R. N. Clarke, W. Cleland, J. C. Clemens, C. Clement, Y. Coadou, M. Cobal, A. Coccaro, J. Cochran, L. Coffey, J. G. Cogan, J. Coggeshall, B. Cole, S. Cole, A. P. Colijn, J. Collot, T. Colombo, G. Colon, G. Compostella, P. Conde Muiño, E. Coniavitis, M. C. Conidi, S. H. Connell, I. A. Connelly, S. M. Consonni, V. Consorti, S. Constantinescu, C. Conta, G. Conti, F. Conventi, M. Cooke, B. D. Cooper, A. M. Cooper-Sarkar, N. J. Cooper-Smith, K. Copic, T. Cornelissen, M. Corradi, F. Corriveau, A. Corso-Radu, A. Cortes-Gonzalez, G. Cortiana, G. Costa, M. J. Costa, D. Costanzo, D. Côté, G. Cottin, G. Cowan, B. E. Cox, K. Cranmer, G. Cree, S. Crépé-Renaudin, F. Crescioli, W. A. Cribbs, M. Crispin Ortuzar, M. Cristinziani, V. Croft, G. Crosetti, C.-M. Cuciuc, T. Cuhadar Donszelmann, J. Cummings, M. Curatolo, C. Cuthbert, H. Czirr, P. Czodrowski, Z. Czyczula, S. D’Auria, M. D’Onofrio, M. J. Da Cunha Sargedas De Sousa, C. Da Via, W. Dabrowski, A. Dafinca, T. Dai, O. Dale, F. Dallaire, C. Dallapiccola, M. Dam, A. C. Daniells, M. Dano Hoffmann, V. Dao, G. Darbo, S. Darmora, J. A. Dassoulas, A. Dattagupta, W. Davey, C. David, T. Davidek, E. Davies, M. Davies, O. Davignon, A. R. Davison, P. Davison, Y. Davygora, E. Dawe, I. Dawson, R. K. Daya-Ishmukhametova, K. De, R. de Asmundis, S. De Castro, S. De Cecco, N. De Groot, P. de Jong, H. De la Torre, F. De Lorenzi, L. De Nooij, D. De Pedis, A. De Salvo, U. De Sanctis, A. De Santo, J. B. De Vivie De Regie, W. J. Dearnaley, R. Debbe, C. Debenedetti, B. Dechenaux, D. V. Dedovich, I. Deigaard, J. Del Peso, T. Del Prete, F. Deliot, C. M. Delitzsch, M. Deliyergiyev, A. Dell’Acqua, L. Dell’Asta, M. Dell’Orso, M. Della Pietra, D. della Volpe, M. Delmastro, P. A. Delsart, C. Deluca, S. Demers, M. Demichev, A. Demilly, S. P. Denisov, D. Derendarz, J. E. Derkaoui, F. Derue, P. Dervan, K. Desch, C. Deterre, P. O. Deviveiros, A. Dewhurst, S. Dhaliwal, A. Di Ciaccio, L. Di Ciaccio, A. Di Domenico, C. Di Donato, A. Di Girolamo, B. Di Girolamo, A. Di Mattia, B. Di Micco, R. Di Nardo, A. Di Simone, R. Di Sipio, D. Di Valentino, F. A. Dias, M. A. Diaz, E. B. Diehl, J. Dietrich, T. A. Dietzsch, S. Diglio, A. Dimitrievska, J. Dingfelder, C. Dionisi, P. Dita, S. Dita, F. Dittus, F. Djama, T. Djobava, M. A. B. do Vale, A. Do Valle Wemans, T. K. O. Doan, D. Dobos, C. Doglioni, T. Doherty, T. Dohmae, J. Dolejsi, Z. Dolezal, B. A. Dolgoshein, M. Donadelli, S. Donati, P. Dondero, J. Donini, J. Dopke, A. Doria, M. T. Dova, A. T. Doyle, M. Dris, J. Dubbert, S. Dube, E. Dubreuil, E. Duchovni, G. Duckeck, O. A. Ducu, D. Duda, A. Dudarev, F. Dudziak, L. Duflot, L. Duguid, M. Dührssen, M. Dunford, H. Duran Yildiz, M. Düren, A. Durglishvili, M. Dwuznik, M. Dyndal, J. Ebke, W. Edson, N. C. Edwards, W. Ehrenfeld, T. Eifert, G. Eigen, K. Einsweiler, T. Ekelof, M. El Kacimi, M. Ellert, S. Elles, F. Ellinghaus, N. Ellis, J. Elmsheuser, M. Elsing, D. Emeliyanov, Y. Enari, O. C. Endner, M. Endo, R. Engelmann, J. Erdmann, A. Ereditato, D. Eriksson, G. Ernis, J. Ernst, M. Ernst, J. Ernwein, D. Errede, S. Errede, E. Ertel, M. Escalier, H. Esch, C. Escobar, B. Esposito, A. I. Etienvre, E. Etzion, H. Evans, A. Ezhilov, L. Fabbri, G. Facini, R. M. Fakhrutdinov, S. Falciano, R. J. Falla, J. Faltova, Y. Fang, M. Fanti, A. Farbin, A. Farilla, T. Farooque, S. Farrell, S. M. Farrington, P. Farthouat, F. Fassi, P. Fassnacht, D. Fassouliotis, A. Favareto, L. Fayard, P. Federic, O. L. Fedin, W. Fedorko, M. Fehling-Kaschek, S. Feigl, L. Feligioni, C. Feng, E. J. Feng, H. Feng, A. B. Fenyuk, S. Fernandez Perez, S. Ferrag, J. Ferrando, A. Ferrari, P. Ferrari, R. Ferrari, D. E. Ferreira de Lima, A. Ferrer, D. Ferrere, C. Ferretti, A. Ferretto Parodi, M. Fiascaris, F. Fiedler, A. Filipčič, M. Filipuzzi, F. Filthaut, M. Fincke-Keeler, K. D. Finelli, M. C. N. Fiolhais, L. Fiorini, A. Firan, A. Fischer, J. Fischer, W. C. Fisher, E. A. Fitzgerald, M. Flechl, I. Fleck, P. Fleischmann, S. Fleischmann, G. T. Fletcher, G. Fletcher, T. Flick, A. Floderus, L. R. Flores Castillo, A. C. Florez Bustos, M. J. Flowerdew, A. Formica, A. Forti, D. Fortin, D. Fournier, H. Fox, S. Fracchia, P. Francavilla, M. Franchini, S. Franchino, D. Francis, L. Franconi, M. Franklin, S. Franz, M. Fraternali, S. T. French, C. Friedrich, F. Friedrich, D. Froidevaux, J. A. Frost, C. Fukunaga, E. Fullana Torregrosa, B. G. Fulsom, J. Fuster, C. Gabaldon, O. Gabizon, A. Gabrielli, A. Gabrielli, S. Gadatsch, S. Gadomski, G. Gagliardi, P. Gagnon, C. Galea, B. Galhardo, E. J. Gallas, V. Gallo, B. J. Gallop, P. Gallus, G. Galster, K. K. Gan, J. Gao, Y. S. Gao, F. M. Garay Walls, F. Garberson, C. García, J. E. García Navarro, M. Garcia-Sciveres, R. W. Gardner, N. Garelli, V. Garonne, C. Gatti, G. Gaudio, B. Gaur, L. Gauthier, P. Gauzzi, I. L. Gavrilenko, C. Gay, G. Gaycken, E. N. Gazis, P. Ge, Z. Gecse, C. N. P. Gee, D. A. A. Geerts, Ch. Geich-Gimbel, K. Gellerstedt, C. Gemme, A. Gemmell, M. H. Genest, S. Gentile, M. George, S. George, D. Gerbaudo, A. Gershon, H. Ghazlane, N. Ghodbane, B. Giacobbe, S. Giagu, V. Giangiobbe, P. Giannetti, F. Gianotti, B. Gibbard, S. M. Gibson, M. Gilchriese, T. P. S. Gillam, D. Gillberg, G. Gilles, D. M. Gingrich, N. Giokaris, M. P. Giordani, R. Giordano, F. M. Giorgi, F. M. Giorgi, P. F. Giraud, D. Giugni, C. Giuliani, M. Giulini, B. K. Gjelsten, S. Gkaitatzis, I. Gkialas, L. K. Gladilin, C. Glasman, J. Glatzer, P. C. F. Glaysher, A. Glazov, G. L. Glonti, M. Goblirsch-Kolb, J. R. Goddard, J. Godfrey, J. Godlewski, C. Goeringer, S. Goldfarb, T. Golling, D. Golubkov, A. Gomes, L. S. Gomez Fajardo, R. Gonçalo, J. Goncalves Pinto Firmino Da Costa, L. Gonella, S. González de la Hoz, G. Gonzalez Parra, S. Gonzalez-Sevilla, L. Goossens, P. A. Gorbounov, H. A. Gordon, I. Gorelov, B. Gorini, E. Gorini, A. Gorišek, E. Gornicki, A. T. Goshaw, C. Gössling, M. I. Gostkin, M. Gouighri, D. Goujdami, M. P. Goulette, A. G. Goussiou, C. Goy, S. Gozpinar, H. M. X. Grabas, L. Graber, I. Grabowska-Bold, P. Grafström, K-J. Grahn, J. Gramling, E. Gramstad, S. Grancagnolo, V. Grassi, V. Gratchev, H. M. Gray, E. Graziani, O. G. Grebenyuk, Z. D. Greenwood, K. Gregersen, I. M. Gregor, P. Grenier, J. Griffiths, A. A. Grillo, K. Grimm, S. Grinstein, Ph. Gris, Y. V. Grishkevich, J.-F. Grivaz, J. P. Grohs, A. Grohsjean, E. Gross, J. Grosse-Knetter, G. C. Grossi, J. Groth-Jensen, Z. J. Grout, L. Guan, F. Guescini, D. Guest, O. Gueta, C. Guicheney, E. Guido, T. Guillemin, S. Guindon, U. Gul, C. Gumpert, J. Gunther, J. Guo, S. Gupta, P. Gutierrez, N. G. Gutierrez Ortiz, C. Gutschow, N. Guttman, C. Guyot, C. Gwenlan, C. B. Gwilliam, A. Haas, C. Haber, H. K. Hadavand, N. Haddad, P. Haefner, S. Hageböck, Z. Hajduk, H. Hakobyan, M. Haleem, D. Hall, G. Halladjian, K. Hamacher, P. Hamal, K. Hamano, M. Hamer, A. Hamilton, S. Hamilton, G. N. Hamity, P. G. Hamnett, L. Han, K. Hanagaki, K. Hanawa, M. Hance, P. Hanke, R. Hanna, J. B. Hansen, J. D. Hansen, P. H. Hansen, K. Hara, A. S. Hard, T. Harenberg, F. Hariri, S. Harkusha, D. Harper, R. D. Harrington, O. M. Harris, P. F. Harrison, F. Hartjes, M. Hasegawa, S. Hasegawa, Y. Hasegawa, A. Hasib, S. Hassani, S. Haug, M. Hauschild, R. Hauser, M. Havranek, C. M. Hawkes, R. J. Hawkings, A. D. Hawkins, T. Hayashi, D. Hayden, C. P. Hays, H. S. Hayward, S. J. Haywood, S. J. Head, T. Heck, V. Hedberg, L. Heelan, S. Heim, T. Heim, B. Heinemann, L. Heinrich, J. Hejbal, L. Helary, C. Heller, M. Heller, S. Hellman, D. Hellmich, C. Helsens, J. Henderson, R. C. W. Henderson, Y. Heng, C. Hengler, A. Henrichs, A. M. Henriques Correia, S. Henrot-Versille, C. Hensel, G. H. Herbert, Y. Hernández Jiménez, R. Herrberg-Schubert, G. Herten, R. Hertenberger, L. Hervas, G. G. Hesketh, N. P. Hessey, R. Hickling, E. Higón-Rodriguez, E. Hill, J. C. Hill, K. H. Hiller, S. Hillert, S. J. Hillier, I. Hinchliffe, E. Hines, M. Hirose, D. Hirschbuehl, J. Hobbs, N. Hod, M. C. Hodgkinson, P. Hodgson, A. Hoecker, M. R. Hoeferkamp, F. Hoenig, J. Hoffman, D. Hoffmann, J. I. Hofmann, M. Hohlfeld, T. R. Holmes, T. M. Hong, L. Hooft van Huysduynen, Y. Horii, J-Y. Hostachy, S. Hou, A. Hoummada, J. Howard, J. Howarth, M. Hrabovsky, I. Hristova, J. Hrivnac, T. Hryn’ova, C. Hsu, P. J. Hsu, S.-C. Hsu, D. Hu, X. Hu, Y. Huang, Z. Hubacek, F. Hubaut, F. Huegging, T. B. Huffman, E. W. Hughes, G. Hughes, M. Huhtinen, T. A. Hülsing, M. Hurwitz, N. Huseynov, J. Huston, J. Huth, G. Iacobucci, G. Iakovidis, I. Ibragimov, L. Iconomidou-Fayard, E. Ideal, P. Iengo, O. Igonkina, T. Iizawa, Y. Ikegami, K. Ikematsu, M. Ikeno, Y. Ilchenko, D. Iliadis, N. Ilic, Y. Inamaru, T. Ince, P. Ioannou, M. Iodice, K. Iordanidou, V. Ippolito, A. Irles Quiles, C. Isaksson, M. Ishino, M. Ishitsuka, R. Ishmukhametov, C. Issever, S. Istin, J. M. Iturbe Ponce, R. Iuppa, J. Ivarsson, W. Iwanski, H. Iwasaki, J. M. Izen, V. Izzo, B. Jackson, M. Jackson, P. Jackson, M. R. Jaekel, V. Jain, K. Jakobs, S. Jakobsen, T. Jakoubek, J. Jakubek, D. O. Jamin, D. K. Jana, E. Jansen, H. Jansen, J. Janssen, M. Janus, G. Jarlskog, N. Javadov, T. Javůrek, L. Jeanty, J. Jejelava, G.-Y. Jeng, D. Jennens, P. Jenni, J. Jentzsch, C. Jeske, S. Jézéquel, H. Ji, J. Jia, Y. Jiang, M. Jimenez Belenguer, S. Jin, A. Jinaru, O. Jinnouchi, M. D. Joergensen, K. E. Johansson, P. Johansson, K. A. Johns, K. Jon-And, G. Jones, R. W. L. Jones, T. J. Jones, J. Jongmanns, P. M. Jorge, K. D. Joshi, J. Jovicevic, X. Ju, C. A. Jung, R. M. Jungst, P. Jussel, A. Juste Rozas, M. Kaci, A. Kaczmarska, M. Kado, H. Kagan, M. Kagan, E. Kajomovitz, C. W. Kalderon, S. Kama, A. Kamenshchikov, N. Kanaya, M. Kaneda, S. Kaneti, V. A. Kantserov, J. Kanzaki, B. Kaplan, A. Kapliy, D. Kar, K. Karakostas, N. Karastathis, M. Karnevskiy, S. N. Karpov, Z. M. Karpova, K. Karthik, V. Kartvelishvili, A. N. Karyukhin, L. Kashif, G. Kasieczka, R. D. Kass, A. Kastanas, Y. Kataoka, A. Katre, J. Katzy, V. Kaushik, K. Kawagoe, T. Kawamoto, G. Kawamura, S. Kazama, V. F. Kazanin, M. Y. Kazarinov, R. Keeler, R. Kehoe, M. Keil, J. S. Keller, J. J. Kempster, H. Keoshkerian, O. Kepka, B. P. Kerševan, S. Kersten, K. Kessoku, J. Keung, F. Khalil-zada, H. Khandanyan, A. Khanov, A. Khodinov, A. Khomich, T. J. Khoo, G. Khoriauli, A. Khoroshilov, V. Khovanskiy, E. Khramov, J. Khubua, H. Y. Kim, H. Kim, S. H. Kim, N. Kimura, O. Kind, B. T. King, M. King, R. S. B. King, S. B. King, J. Kirk, A. E. Kiryunin, T. Kishimoto, D. Kisielewska, F. Kiss, T. Kittelmann, K. Kiuchi, E. Kladiva, M. Klein, U. Klein, K. Kleinknecht, P. Klimek, A. Klimentov, R. Klingenberg, J. A. Klinger, T. Klioutchnikova, P. F. Klok, E.-E. Kluge, P. Kluit, S. Kluth, E. Kneringer, E. B. F. G. Knoops, A. Knue, D. Kobayashi, T. Kobayashi, M. Kobel, M. Kocian, P. Kodys, P. Koevesarki, T. Koffas, E. Koffeman, L. A. Kogan, S. Kohlmann, Z. Kohout, T. Kohriki, T. Koi, H. Kolanoski, I. Koletsou, J. Koll, A. A. Komar, Y. Komori, T. Kondo, N. Kondrashova, K. Köneke, A. C. König, S. König, T. Kono, R. Konoplich, N. Konstantinidis, R. Kopeliansky, S. Koperny, L. Köpke, A. K. Kopp, K. Korcyl, K. Kordas, A. Korn, A. A. Korol, I. Korolkov, E. V. Korolkova, V. A. Korotkov, O. Kortner, S. Kortner, V. V. Kostyukhin, V. M. Kotov, A. Kotwal, C. Kourkoumelis, V. Kouskoura, A. Koutsman, R. Kowalewski, T. Z. Kowalski, W. Kozanecki, A. S. Kozhin, V. Kral, V. A. Kramarenko, G. Kramberger, D. Krasnopevtsev, M. W. Krasny, A. Krasznahorkay, J. K. Kraus, A. Kravchenko, S. Kreiss, M. Kretz, J. Kretzschmar, K. Kreutzfeldt, P. Krieger, K. Kroeninger, H. Kroha, J. Kroll, J. Kroseberg, J. Krstic, U. Kruchonak, H. Krüger, T. Kruker, N. Krumnack, Z. V. Krumshteyn, A. Kruse, M. C. Kruse, M. Kruskal, T. Kubota, S. Kuday, S. Kuehn, A. Kugel, A. Kuhl, T. Kuhl, V. Kukhtin, Y. Kulchitsky, S. Kuleshov, M. Kuna, J. Kunkle, A. Kupco, H. Kurashige, Y. A. Kurochkin, R. Kurumida, V. Kus, E. S. Kuwertz, M. Kuze, J. Kvita, A. La Rosa, L. La Rotonda, C. Lacasta, F. Lacava, J. Lacey, H. Lacker, D. Lacour, V. R. Lacuesta, E. Ladygin, R. Lafaye, B. Laforge, T. Lagouri, S. Lai, H. Laier, L. Lambourne, S. Lammers, C. L. Lampen, W. Lampl, E. Lançon, U. Landgraf, M. P. J. Landon, V. S. Lang, A. J. Lankford, F. Lanni, K. Lantzsch, S. Laplace, C. Lapoire, J. F. Laporte, T. Lari, M. Lassnig, P. Laurelli, W. Lavrijsen, A. T. Law, P. Laycock, O. Le Dortz, E. Le Guirriec, E. Le Menedeu, T. LeCompte, F. Ledroit-Guillon, C. A. Lee, H. Lee, J. S. H. Lee, S. C. Lee, L. Lee, G. Lefebvre, M. Lefebvre, F. Legger, C. Leggett, A. Lehan, M. Lehmacher, G. Lehmann Miotto, X. Lei, W. A. Leight, A. Leisos, A. G. Leister, M. A. L. Leite, R. Leitner, D. Lellouch, B. Lemmer, K. J. C. Leney, T. Lenz, G. Lenzen, B. Lenzi, R. Leone, S. Leone, K. Leonhardt, C. Leonidopoulos, S. Leontsinis, C. Leroy, C. G. Lester, C. M. Lester, M. Levchenko, J. Levêque, D. Levin, L. J. Levinson, M. Levy, A. Lewis, G. H. Lewis, A. M. Leyko, M. Leyton, B. Li, B. Li, H. Li, H. L. Li, L. Li, L. Li, S. Li, Y. Li, Z. Liang, H. Liao, B. Liberti, P. Lichard, K. Lie, J. Liebal, W. Liebig, C. Limbach, A. Limosani, S. C. Lin, T. H. Lin, F. Linde, B. E. Lindquist, J. T. Linnemann, E. Lipeles, A. Lipniacka, M. Lisovyi, T. M. Liss, D. Lissauer, A. Lister, A. M. Litke, B. Liu, D. Liu, J. B. Liu, K. Liu, L. Liu, M. Liu, M. Liu, Y. Liu, M. Livan, S. S. A. Livermore, A. Lleres, J. Llorente Merino, S. L. Lloyd, F. Lo Sterzo, E. Lobodzinska, P. Loch, W. S. Lockman, T. Loddenkoetter, F. K. Loebinger, A. E. Loevschall-Jensen, A. Loginov, T. Lohse, K. Lohwasser, M. Lokajicek, V. P. Lombardo, B. A. Long, J. D. Long, R. E. Long, L. Lopes, D. Lopez Mateos, B. Lopez Paredes, I. Lopez Paz, J. Lorenz, N. Lorenzo Martinez, M. Losada, P. Loscutoff, X. Lou, A. Lounis, J. Love, P. A. Love, A. J. Lowe, F. Lu, N. Lu, H. J. Lubatti, C. Luci, A. Lucotte, F. Luehring, W. Lukas, L. Luminari, O. Lundberg, B. Lund-Jensen, M. Lungwitz, D. Lynn, R. Lysak, E. Lytken, H. Ma, L. L. Ma, G. Maccarrone, A. Macchiolo, J. Machado Miguens, D. Macina, D. Madaffari, R. Madar, H. J. Maddocks, W. F. Mader, A. Madsen, M. Maeno, T. Maeno, E. Magradze, K. Mahboubi, J. Mahlstedt, S. Mahmoud, C. Maiani, C. Maidantchik, A. A. Maier, A. Maio, S. Majewski, Y. Makida, N. Makovec, P. Mal, B. Malaescu, Pa. Malecki, V. P. Maleev, F. Malek, U. Mallik, D. Malon, C. Malone, S. Maltezos, V. M. Malyshev, S. Malyukov, J. Mamuzic, B. Mandelli, L. Mandelli, I. Mandić, R. Mandrysch, J. Maneira, A. Manfredini, L. Manhaes de Andrade Filho, J. A. Manjarres Ramos, A. Mann, P. M. Manning, A. Manousakis-Katsikakis, B. Mansoulie, R. Mantifel, L. Mapelli, L. March, J. F. Marchand, G. Marchiori, M. Marcisovsky, C. P. Marino, M. Marjanovic, C. N. Marques, F. Marroquim, S. P. Marsden, Z. Marshall, L. F. Marti, S. Marti-Garcia, B. Martin, B. Martin, T. A. Martin, V. J. Martin, B. Martin dit Latour, H. Martinez, M. Martinez, S. Martin-Haugh, A. C. Martyniuk, M. Marx, F. Marzano, A. Marzin, L. Masetti, T. Mashimo, R. Mashinistov, J. Masik, A. L. Maslennikov, I. Massa, L. Massa, N. Massol, P. Mastrandrea, A. Mastroberardino, T. Masubuchi, P. Mättig, J. Mattmann, J. Maurer, S. J. Maxfield, D. A. Maximov, R. Mazini, L. Mazzaferro, G. Mc Goldrick, S. P. Mc Kee, A. McCarn, R. L. McCarthy, T. G. McCarthy, N. A. McCubbin, K. W. McFarlane, J. A. Mcfayden, G. Mchedlidze, S. J. McMahon, R. A. McPherson, A. Meade, J. Mechnich, M. Medinnis, S. Meehan, S. Mehlhase, A. Mehta, K. Meier, C. Meineck, B. Meirose, C. Melachrinos, B. R. Mellado Garcia, F. Meloni, A. Mengarelli, S. Menke, E. Meoni, K. M. Mercurio, S. Mergelmeyer, N. Meric, P. Mermod, L. Merola, C. Meroni, F. S. Merritt, H. Merritt, A. Messina, J. Metcalfe, A. S. Mete, C. Meyer, C. Meyer, J-P. Meyer, J. Meyer, R. P. Middleton, S. Migas, L. Mijović, G. Mikenberg, M. Mikestikova, M. Mikuž, A. Milic, D. W. Miller, C. Mills, A. Milov, D. A. Milstead, D. Milstein, A. A. Minaenko, I. A. Minashvili, A. I. Mincer, B. Mindur, M. Mineev, Y. Ming, L. M. Mir, G. Mirabelli, T. Mitani, J. Mitrevski, V. A. Mitsou, S. Mitsui, A. Miucci, P. S. Miyagawa, J. U. Mjörnmark, T. Moa, K. Mochizuki, S. Mohapatra, W. Mohr, S. Molander, R. Moles-Valls, K. Mönig, C. Monini, J. Monk, E. Monnier, J. Montejo Berlingen, F. Monticelli, S. Monzani, R. W. Moore, A. Moraes, N. Morange, D. Moreno, M. Moreno Llácer, P. Morettini, M. Morgenstern, M. Morii, S. Moritz, A. K. Morley, G. Mornacchi, J. D. Morris, L. Morvaj, H. G. Moser, M. Mosidze, J. Moss, K. Motohashi, R. Mount, E. Mountricha, S. V. Mouraviev, E. J. W. Moyse, S. Muanza, R. D. Mudd, F. Mueller, J. Mueller, K. Mueller, T. Mueller, T. Mueller, D. Muenstermann, Y. Munwes, J. A. Murillo Quijada, W. J. Murray, H. Musheghyan, E. Musto, A. G. Myagkov, M. Myska, O. Nackenhorst, J. Nadal, K. Nagai, R. Nagai, Y. Nagai, K. Nagano, A. Nagarkar, Y. Nagasaka, M. Nagel, A. M. Nairz, Y. Nakahama, K. Nakamura, T. Nakamura, I. Nakano, H. Namasivayam, G. Nanava, R. Narayan, T. Nattermann, T. Naumann, G. Navarro, R. Nayyar, H. A. Neal, P. Yu. Nechaeva, T. J. Neep, P. D. Nef, A. Negri, G. Negri, M. Negrini, S. Nektarijevic, A. Nelson, T. K. Nelson, S. Nemecek, P. Nemethy, A. A. Nepomuceno, M. Nessi, M. S. Neubauer, M. Neumann, R. M. Neves, P. Nevski, P. R. Newman, D. H. Nguyen, R. B. Nickerson, R. Nicolaidou, B. Nicquevert, J. Nielsen, N. Nikiforou, A. Nikiforov, V. Nikolaenko, I. Nikolic-Audit, K. Nikolics, K. Nikolopoulos, P. Nilsson, Y. Ninomiya, A. Nisati, R. Nisius, T. Nobe, L. Nodulman, M. Nomachi, I. Nomidis, S. Norberg, M. Nordberg, O. Novgorodova, S. Nowak, M. Nozaki, L. Nozka, K. Ntekas, G. Nunes Hanninger, T. Nunnemann, E. Nurse, F. Nuti, B. J. O’Brien, F. O’Grady, D. C. O’Neil, V. O’Shea, F. G. Oakham, H. Oberlack, T. Obermann, J. Ocariz, A. Ochi, M. I. Ochoa, S. Oda, S. Odaka, H. Ogren, A. Oh, S. H. Oh, C. C. Ohm, H. Ohman, W. Okamura, H. Okawa, Y. Okumura, T. Okuyama, A. Olariu, A. G. Olchevski, S. A. Olivares Pino, D. Oliveira Damazio, E. Oliver Garcia, A. Olszewski, J. Olszowska, A. Onofre, P. U. E. Onyisi, C. J. Oram, M. J. Oreglia, Y. Oren, D. Orestano, N. Orlando, C. Oropeza Barrera, R. S. Orr, B. Osculati, R. Ospanov, G. Otero y Garzon, H. Otono, M. Ouchrif, E. A. Ouellette, F. Ould-Saada, A. Ouraou, K. P. Oussoren, Q. Ouyang, A. Ovcharova, M. Owen, V. E. Ozcan, N. Ozturk, K. Pachal, A. Pacheco Pages, C. Padilla Aranda, M. Pagáčová, S. Pagan Griso, E. Paganis, C. Pahl, F. Paige, P. Pais, K. Pajchel, G. Palacino, S. Palestini, M. Palka, D. Pallin, A. Palma, J. D. Palmer, Y. B. Pan, E. Panagiotopoulou, J. G. Panduro Vazquez, P. Pani, N. Panikashvili, S. Panitkin, D. Pantea, L. Paolozzi, Th. D. Papadopoulou, K. Papageorgiou, A. Paramonov, D. Paredes Hernandez, M. A. Parker, F. Parodi, J. A. Parsons, U. Parzefall, E. Pasqualucci, S. Passaggio, A. Passeri, F. Pastore, Fr. Pastore, G. Pásztor, S. Pataraia, N. D. Patel, J. R. Pater, S. Patricelli, T. Pauly, J. Pearce, M. Pedersen, S. Pedraza Lopez, R. Pedro, S. V. Peleganchuk, D. Pelikan, H. Peng, B. Penning, J. Penwell, D. V. Perepelitsa, E. Perez Codina, M. T. Pérez García-Estañ, V. Perez Reale, L. Perini, H. Pernegger, R. Perrino, R. Peschke, V. D. Peshekhonov, K. Peters, R. F. Y. Peters, B. A. Petersen, T. C. Petersen, E. Petit, A. Petridis, C. Petridou, E. Petrolo, F. Petrucci, N. E. Pettersson, R. Pezoa, P. W. Phillips, G. Piacquadio, E. Pianori, A. Picazio, E. Piccaro, M. Piccinini, R. Piegaia, D. T. Pignotti, J. E. Pilcher, A. D. Pilkington, J. Pina, M. Pinamonti, A. Pinder, J. L. Pinfold, A. Pingel, B. Pinto, S. Pires, M. Pitt, C. Pizio, L. Plazak, M.-A. Pleier, V. Pleskot, E. Plotnikova, P. Plucinski, S. Poddar, F. Podlyski, R. Poettgen, L. Poggioli, D. Pohl, M. Pohl, G. Polesello, A. Policicchio, R. Polifka, A. Polini, C. S. Pollard, V. Polychronakos, K. Pommès, L. Pontecorvo, B. G. Pope, G. A. Popeneciu, D. S. Popovic, A. Poppleton, X. Portell Bueso, S. Pospisil, K. Potamianos, I. N. Potrap, C. J. Potter, C. T. Potter, G. Poulard, J. Poveda, V. Pozdnyakov, P. Pralavorio, A. Pranko, S. Prasad, R. Pravahan, S. Prell, D. Price, J. Price, L. E. Price, D. Prieur, M. Primavera, M. Proissl, K. Prokofiev, F. Prokoshin, E. Protopapadaki, S. Protopopescu, J. Proudfoot, M. Przybycien, H. Przysiezniak, E. Ptacek, D. Puddu, E. Pueschel, D. Puldon, M. Purohit, P. Puzo, J. Qian, G. Qin, Y. Qin, A. Quadt, D. R. Quarrie, W. B. Quayle, M. Queitsch-Maitland, D. Quilty, A. Qureshi, V. Radeka, V. Radescu, S. K. Radhakrishnan, P. Radloff, P. Rados, F. Ragusa, G. Rahal, S. Rajagopalan, M. Rammensee, A. S. Randle-Conde, C. Rangel-Smith, K. Rao, F. Rauscher, T. C. Rave, T. Ravenscroft, M. Raymond, A. L. Read, N. P. Readioff, D. M. Rebuzzi, A. Redelbach, G. Redlinger, R. Reece, K. Reeves, L. Rehnisch, H. Reisin, M. Relich, C. Rembser, H. Ren, Z. L. Ren, A. Renaud, M. Rescigno, S. Resconi, O. L. Rezanova, P. Reznicek, R. Rezvani, R. Richter, M. Ridel, P. Rieck, J. Rieger, M. Rijssenbeek, A. Rimoldi, L. Rinaldi, E. Ritsch, I. Riu, F. Rizatdinova, E. Rizvi, S. H. Robertson, A. Robichaud-Veronneau, D. Robinson, J. E. M. Robinson, A. Robson, C. Roda, L. Rodrigues, S. Roe, O. Røhne, S. Rolli, A. Romaniouk, M. Romano, E. Romero Adam, N. Rompotis, M. Ronzani, L. Roos, E. Ros, S. Rosati, K. Rosbach, M. Rose, P. Rose, P. L. Rosendahl, O. Rosenthal, V. Rossetti, E. Rossi, L. P. Rossi, R. Rosten, M. Rotaru, I. Roth, J. Rothberg, D. Rousseau, C. R. Royon, A. Rozanov, Y. Rozen, X. Ruan, F. Rubbo, I. Rubinskiy, V. I. Rud, C. Rudolph, M. S. Rudolph, F. Rühr, A. Ruiz-Martinez, Z. Rurikova, N. A. Rusakovich, A. Ruschke, J. P. Rutherfoord, N. Ruthmann, Y. F. Ryabov, M. Rybar, G. Rybkin, N. C. Ryder, A. F. Saavedra, S. Sacerdoti, A. Saddique, I. Sadeh, H. F-W. Sadrozinski, R. Sadykov, F. Safai Tehrani, H. Sakamoto, Y. Sakurai, G. Salamanna, A. Salamon, M. Saleem, D. Salek, P. H. Sales De Bruin, D. Salihagic, A. Salnikov, J. Salt, D. Salvatore, F. Salvatore, A. Salvucci, A. Salzburger, D. Sampsonidis, A. Sanchez, J. Sánchez, V. Sanchez Martinez, H. Sandaker, R. L. Sandbach, H. G. Sander, M. P. Sanders, M. Sandhoff, T. Sandoval, C. Sandoval, R. Sandstroem, D. P. C. Sankey, A. Sansoni, C. Santoni, R. Santonico, H. Santos, I. Santoyo Castillo, K. Sapp, A. Sapronov, J. G. Saraiva, B. Sarrazin, G. Sartisohn, O. Sasaki, Y. Sasaki, G. Sauvage, E. Sauvan, P. Savard, D. O. Savu, C. Sawyer, L. Sawyer, D. H. Saxon, J. Saxon, C. Sbarra, A. Sbrizzi, T. Scanlon, D. A. Scannicchio, M. Scarcella, V. Scarfone, J. Schaarschmidt, P. Schacht, D. Schaefer, R. Schaefer, S. Schaepe, S. Schaetzel, U. Schäfer, A. C. Schaffer, D. Schaile, R. D. Schamberger, V. Scharf, V. A. Schegelsky, D. Scheirich, M. Schernau, M. I. Scherzer, C. Schiavi, J. Schieck, C. Schillo, M. Schioppa, S. Schlenker, E. Schmidt, K. Schmieden, C. Schmitt, C. Schmitt, S. Schmitt, B. Schneider, Y. J. Schnellbach, U. Schnoor, L. Schoeffel, A. Schoening, B. D. Schoenrock, A. L. S. Schorlemmer, M. Schott, D. Schouten, J. Schovancova, S. Schramm, M. Schreyer, C. Schroeder, N. Schuh, M. J. Schultens, H.-C. Schultz-Coulon, H. Schulz, M. Schumacher, B. A. Schumm, Ph. Schune, C. Schwanenberger, A. Schwartzman, Ph. Schwegler, Ph. Schwemling, R. Schwienhorst, J. Schwindling, T. Schwindt, M. Schwoerer, F. G. Sciacca, E. Scifo, G. Sciolla, W. G. Scott, F. Scuri, F. Scutti, J. Searcy, G. Sedov, E. Sedykh, S. C. Seidel, A. Seiden, F. Seifert, J. M. Seixas, G. Sekhniaidze, S. J. Sekula, K. E. Selbach, D. M. Seliverstov, G. Sellers, N. Semprini-Cesari, C. Serfon, L. Serin, L. Serkin, T. Serre, R. Seuster, H. Severini, T. Sfiligoj, F. Sforza, A. Sfyrla, E. Shabalina, M. Shamim, L. Y. Shan, R. Shang, J. T. Shank, M. Shapiro, P. B. Shatalov, K. Shaw, C. Y. Shehu, P. Sherwood, L. Shi, S. Shimizu, C. O. Shimmin, M. Shimojima, M. Shiyakova, A. Shmeleva, M. J. Shochet, D. Short, S. Shrestha, E. Shulga, M. A. Shupe, S. Shushkevich, P. Sicho, O. Sidiropoulou, D. Sidorov, A. Sidoti, F. Siegert, Dj. Sijacki, J. Silva, Y. Silver, D. Silverstein, S. B. Silverstein, V. Simak, O. Simard, Lj. Simic, S. Simion, E. Simioni, B. Simmons, R. Simoniello, M. Simonyan, P. Sinervo, N. B. Sinev, V. Sipica, G. Siragusa, A. Sircar, A. N. Sisakyan, S. Yu. Sivoklokov, J. Sjölin, T. B. Sjursen, H. P. Skottowe, K. Yu. Skovpen, P. Skubic, M. Slater, T. Slavicek, K. Sliwa, V. Smakhtin, B. H. Smart, L. Smestad, S. Yu. Smirnov, Y. Smirnov, L. N. Smirnova, O. Smirnova, K. M. Smith, M. Smizanska, K. Smolek, A. A. Snesarev, G. Snidero, S. Snyder, R. Sobie, F. Socher, A. Soffer, D. A. Soh, C. A. Solans, M. Solar, J. Solc, E. Yu. Soldatov, U. Soldevila, A. A. Solodkov, A. Soloshenko, O. V. Solovyanov, V. Solovyev, P. Sommer, H. Y. Song, N. Soni, A. Sood, A. Sopczak, B. Sopko, V. Sopko, V. Sorin, M. Sosebee, R. Soualah, P. Soueid, A. M. Soukharev, D. South, S. Spagnolo, F. Spanò, W. R. Spearman, F. Spettel, R. Spighi, G. Spigo, M. Spousta, T. Spreitzer, B. Spurlock, R. D. St. Denis, S. Staerz, J. Stahlman, R. Stamen, E. Stanecka, R. W. Stanek, C. Stanescu, M. Stanescu-Bellu, M. M. Stanitzki, S. Stapnes, E. A. Starchenko, J. Stark, P. Staroba, P. Starovoitov, R. Staszewski, P. Stavina, P. Steinberg, B. Stelzer, H. J. Stelzer, O. Stelzer-Chilton, H. Stenzel, S. Stern, G. A. Stewart, J. A. Stillings, M. C. Stockton, M. Stoebe, G. Stoicea, P. Stolte, S. Stonjek, A. R. Stradling, A. Straessner, M. E. Stramaglia, J. Strandberg, S. Strandberg, A. Strandlie, E. Strauss, M. Strauss, P. Strizenec, R. Ströhmer, D. M. Strom, R. Stroynowski, S. A. Stucci, B. Stugu, N. A. Styles, D. Su, J. Su, R. Subramaniam, A. Succurro, Y. Sugaya, C. Suhr, M. Suk, V. V. Sulin, S. Sultansoy, T. Sumida, S. Sun, X. Sun, J. E. Sundermann, K. Suruliz, G. Susinno, M. R. Sutton, Y. Suzuki, M. Svatos, S. Swedish, M. Swiatlowski, I. Sykora, T. Sykora, D. Ta, C. Taccini, K. Tackmann, J. Taenzer, A. Taffard, R. Tafirout, N. Taiblum, H. Takai, R. Takashima, H. Takeda, T. Takeshita, Y. Takubo, M. Talby, A. A. Talyshev, J. Y. C. Tam, K. G. Tan, J. Tanaka, R. Tanaka, S. Tanaka, S. Tanaka, A. J. Tanasijczuk, B. B. Tannenwald, N. Tannoury, S. Tapprogge, S. Tarem, F. Tarrade, G. F. Tartarelli, P. Tas, M. Tasevsky, T. Tashiro, E. Tassi, A. Tavares Delgado, Y. Tayalati, F. E. Taylor, G. N. Taylor, W. Taylor, F. A. Teischinger, M. Teixeira Dias Castanheira, P. Teixeira-Dias, K. K. Temming, H. Ten Kate, P. K. Teng, J. J. Teoh, S. Terada, K. Terashi, J. Terron, S. Terzo, M. Testa, R. J. Teuscher, J. Therhaag, T. Theveneaux-Pelzer, J. P. Thomas, J. Thomas-Wilsker, E. N. Thompson, P. D. Thompson, P. D. Thompson, A. S. Thompson, L. A. Thomsen, E. Thomson, M. Thomson, W. M. Thong, R. P. Thun, F. Tian, M. J. Tibbetts, V. O. Tikhomirov, Yu. A. Tikhonov, S. Timoshenko, E. Tiouchichine, P. Tipton, S. Tisserant, T. Todorov, S. Todorova-Nova, B. Toggerson, J. Tojo, S. Tokár, K. Tokushuku, K. Tollefson, L. Tomlinson, M. Tomoto, L. Tompkins, K. Toms, N. D. Topilin, E. Torrence, H. Torres, E. Torró Pastor, J. Toth, F. Touchard, D. R. Tovey, H. L. Tran, T. Trefzger, L. Tremblet, A. Tricoli, I. M. Trigger, S. Trincaz-Duvoid, M. F. Tripiana, W. Trischuk, B. Trocmé, C. Troncon, M. Trottier-McDonald, M. Trovatelli, P. True, M. Trzebinski, A. Trzupek, C. Tsarouchas, J. C-L. Tseng, P. V. Tsiareshka, D. Tsionou, G. Tsipolitis, N. Tsirintanis, S. Tsiskaridze, V. Tsiskaridze, E. G. Tskhadadze, I. I. Tsukerman, V. Tsulaia, S. Tsuno, D. Tsybychev, A. Tudorache, V. Tudorache, A. N. Tuna, S. A. Tupputi, S. Turchikhin, D. Turecek, I. Turk Cakir, R. Turra, P. M. Tuts, A. Tykhonov, M. Tylmad, M. Tyndel, K. Uchida, I. Ueda, R. Ueno, M. Ughetto, M. Ugland, M. Uhlenbrock, F. Ukegawa, G. Unal, A. Undrus, G. Unel, F. C. Ungaro, Y. Unno, D. Urbaniec, P. Urquijo, G. Usai, A. Usanova, L. Vacavant, V. Vacek, B. Vachon, N. Valencic, S. Valentinetti, A. Valero, L. Valery, S. Valkar, E. Valladolid Gallego, S. Vallecorsa, J. A. Valls Ferrer, W. Van Den Wollenberg, P. C. Van Der Deijl, R. van der Geer, H. van der Graaf, R. Van Der Leeuw, D. van der Ster, N. van Eldik, P. van Gemmeren, J. Van Nieuwkoop, I. van Vulpen, M. C. van Woerden, M. Vanadia, W. Vandelli, R. Vanguri, A. Vaniachine, P. Vankov, F. Vannucci, G. Vardanyan, R. Vari, E. W. Varnes, T. Varol, D. Varouchas, A. Vartapetian, K. E. Varvell, F. Vazeille, T. Vazquez Schroeder, J. Veatch, F. Veloso, S. Veneziano, A. Ventura, D. Ventura, M. Venturi, N. Venturi, A. Venturini, V. Vercesi, M. Verducci, W. Verkerke, J. C. Vermeulen, A. Vest, M. C. Vetterli, O. Viazlo, I. Vichou, T. Vickey, O. E. Vickey Boeriu, G. H. A. Viehhauser, S. Viel, R. Vigne, M. Villa, M. Villaplana Perez, E. Vilucchi, M. G. Vincter, V. B. Vinogradov, J. Virzi, I. Vivarelli, F. Vives Vaque, S. Vlachos, D. Vladoiu, M. Vlasak, A. Vogel, M. Vogel, P. Vokac, G. Volpi, M. Volpi, H. von der Schmitt, H. von Radziewski, E. von Toerne, V. Vorobel, K. Vorobev, M. Vos, R. Voss, J. H. Vossebeld, N. Vranjes, M. Vranjes Milosavljevic, V. Vrba, M. Vreeswijk, T. Vu Anh, R. Vuillermet, I. Vukotic, Z. Vykydal, P. Wagner, W. Wagner, H. Wahlberg, S. Wahrmund, J. Wakabayashi, J. Walder, R. Walker, W. Walkowiak, R. Wall, P. Waller, B. Walsh, C. Wang, C. Wang, F. Wang, H. Wang, H. Wang, J. Wang, J. Wang, K. Wang, R. Wang, S. M. Wang, T. Wang, X. Wang, C. Wanotayaroj, A. Warburton, C. P. Ward, D. R. Wardrope, M. Warsinsky, A. Washbrook, C. Wasicki, P. M. Watkins, A. T. Watson, I. J. Watson, M. F. Watson, G. Watts, S. Watts, B. M. Waugh, S. Webb, M. S. Weber, S. W. Weber, J. S. Webster, A. R. Weidberg, P. Weigell, B. Weinert, J. Weingarten, C. Weiser, H. Weits, P. S. Wells, T. Wenaus, D. Wendland, Z. Weng, T. Wengler, S. Wenig, N. Wermes, M. Werner, P. Werner, M. Wessels, J. Wetter, K. Whalen, A. White, M. J. White, R. White, S. White, D. Whiteson, D. Wicke, F. J. Wickens, W. Wiedenmann, M. Wielers, P. Wienemann, C. Wiglesworth, L. A. M. Wiik-Fuchs, P. A. Wijeratne, A. Wildauer, M. A. Wildt, H. G. Wilkens, J. Z. Will, H. H. Williams, S. Williams, C. Willis, S. Willocq, A. Wilson, J. A. Wilson, I. Wingerter-Seez, F. Winklmeier, B. T. Winter, M. Wittgen, T. Wittig, J. Wittkowski, S. J. Wollstadt, M. W. Wolter, H. Wolters, B. K. Wosiek, J. Wotschack, M. J. Woudstra, K. W. Wozniak, M. Wright, M. Wu, S. L. Wu, X. Wu, Y. Wu, E. Wulf, T. R. Wyatt, B. M. Wynne, S. Xella, M. Xiao, D. Xu, L. Xu, B. Yabsley, S. Yacoob, R. Yakabe, M. Yamada, H. Yamaguchi, Y. Yamaguchi, A. Yamamoto, K. Yamamoto, S. Yamamoto, T. Yamamura, T. Yamanaka, K. Yamauchi, Y. Yamazaki, Z. Yan, H. Yang, H. Yang, U. K. Yang, Y. Yang, S. Yanush, L. Yao, W-M. Yao, Y. Yasu, E. Yatsenko, K. H. Yau Wong, J. Ye, S. Ye, A. L. Yen, E. Yildirim, M. Yilmaz, R. Yoosoofmiya, K. Yorita, R. Yoshida, K. Yoshihara, C. Young, C. J. S. Young, S. Youssef, D. R. Yu, J. Yu, J. M. Yu, J. Yu, L. Yuan, A. Yurkewicz, I. Yusuff, B. Zabinski, R. Zaidan, A. M. Zaitsev, A. Zaman, S. Zambito, L. Zanello, D. Zanzi, C. Zeitnitz, M. Zeman, A. Zemla, K. Zengel, O. Zenin, T. Ženiš, D. Zerwas, G. Zevi della Porta, D. Zhang, F. Zhang, H. Zhang, J. Zhang, L. Zhang, X. Zhang, Z. Zhang, Z. Zhao, A. Zhemchugov, J. Zhong, B. Zhou, L. Zhou, N. Zhou, C. G. Zhu, H. Zhu, J. Zhu, Y. Zhu, X. Zhuang, K. Zhukov, A. Zibell, D. Zieminska, N. I. Zimine, C. Zimmermann, R. Zimmermann, S. Zimmermann, S. Zimmermann, Z. Zinonos, M. Ziolkowski, G. Zobernig, A. Zoccoli, M. zur Nedden, G. Zurzolo, V. Zutshi, L. Zwalinski

**Affiliations:** 1 Department of Physics, University of Adelaide, Adelaide, Australia; 2 Physics Department, SUNY Albany, Albany, NY USA; 3 Department of Physics, University of Alberta, Edmonton, AB Canada; 4 Department of Physics, Ankara University, Ankara, Turkey; Department of Physics, Gazi University, Ankara, Turkey; Division of Physics, TOBB University of Economics and Technology, Ankara, Turkey; Turkish Atomic Energy Authority, Ankara, Turkey; 5 LAPP, CNRS/IN2P3 and Université de Savoie, Annecy-le-Vieux, France; 6 High Energy Physics Division, Argonne National Laboratory, Argonne, IL USA; 7 Department of Physics, University of Arizona, Tucson, AZ USA; 8 Department of Physics, The University of Texas at Arlington, Arlington, TX USA; 9 Physics Department, University of Athens, Athens, Greece; 10 Physics Department, National Technical University of Athens, Zografou, Athens, Greece; 11 Institute of Physics, Azerbaijan Academy of Sciences, Baku, Azerbaijan; 12 Institut de Física d’Altes Energies and Departament de Física de la Universitat Autònoma de Barcelona, Barcelona, Spain; 13 Institute of Physics, University of Belgrade, Belgrade, Serbia; Vinca Institute of Nuclear Sciences, University of Belgrade, Belgrade, Serbia; 14 Department for Physics and Technology, University of Bergen, Bergen, Norway; 15 Physics Division, Lawrence Berkeley National Laboratory and University of California, Berkeley, CA USA; 16 Department of Physics, Humboldt University, Berlin, Germany; 17 Albert Einstein Center for Fundamental Physics and Laboratory for High Energy Physics, University of Bern, Bern, Switzerland; 18 School of Physics and Astronomy, University of Birmingham, Birmingham, UK; 19 Department of Physics, Bogazici University, Istanbul, Turkey; Department of Physics, Dogus University, Istanbul, Turkey; Department of Physics Engineering, Gaziantep University, Gaziantep, Turkey; 20 INFN Sezione di Bologna, Bologna, Italy; Dipartimento di Fisica e Astronomia, Università di Bologna, Bologna, Italy; 21 Physikalisches Institut, University of Bonn, Bonn, Germany; 22 Department of Physics, Boston University, Boston, MA USA; 23 Department of Physics, Brandeis University, Waltham, MA USA; 24 Universidade Federal do Rio De Janeiro COPPE/EE/IF, Rio de Janeiro, Brazil; Federal University of Juiz de Fora (UFJF), Juiz de Fora, Brazil; Federal University of Sao Joao del Rei (UFSJ), Sao Joao del Rei, Brazil; Instituto de Fisica, Universidade de Sao Paulo, São Paulo, Brazil; 25 Physics Department, Brookhaven National Laboratory, Upton, NY USA; 26 National Institute of Physics and Nuclear Engineering, Bucharest, Romania; Physics Department, National Institute for Research and Development of Isotopic and Molecular Technologies, Cluj-Napoca, Romania; University Politehnica Bucharest, Bucharest, Romania; West University in Timisoara, Timisoara, Romania; 27 Departamento de Física, Universidad de Buenos Aires, Buenos Aires, Argentina; 28 Cavendish Laboratory, University of Cambridge, Cambridge, UK; 29 Department of Physics, Carleton University, Ottawa, ON Canada; 30 CERN, Geneva, Switzerland; 31 Enrico Fermi Institute, University of Chicago, Chicago, IL USA; 32 Departamento de Física, Pontificia Universidad Católica de Chile, Santiago, Chile; Departamento de Física, Universidad Técnica Federico Santa María, Valparaiso, Chile; 33 Institute of High Energy Physics, Chinese Academy of Sciences, Beijing, China; Department of Modern Physics, University of Science and Technology of China, Hefei, Anhui, China; Department of Physics, Nanjing University, Nanjing, Jiangsu, China; School of Physics, Shandong University, Jinan, Shandong, China; Physics Department, Shanghai Jiao Tong University, Shanghai, China; 34 Laboratoire de Physique Corpusculaire, Clermont Université and Université Blaise Pascal and CNRS/IN2P3, Clermont-Ferrand, France; 35 Nevis Laboratory, Columbia University, Irvington, NY USA; 36 Niels Bohr Institute, University of Copenhagen, Copenhagen, Denmark; 37 INFN Gruppo Collegato di Cosenza, Laboratori Nazionali di Frascati, Frascati, Italy; Dipartimento di Fisica, Università della Calabria, Rende, Italy; 38 Faculty of Physics and Applied Computer Science, AGH University of Science and Technology, Kraków, Poland; Marian Smoluchowski Institute of Physics, Jagiellonian University, Kraków, Poland; 39 The Henryk Niewodniczanski Institute of Nuclear Physics, Polish Academy of Sciences, Kraków, Poland; 40 Physics Department, Southern Methodist University, Dallas, TX USA; 41 Physics Department, University of Texas at Dallas, Richardson, TX USA; 42 DESY, Hamburg and Zeuthen, Germany; 43 Institut für Experimentelle Physik IV, Technische Universität Dortmund, Dortmund, Germany; 44 Institut für Kern- und Teilchenphysik, Technische Universität Dresden, Dresden, Germany; 45 Department of Physics, Duke University, Durham, NC USA; 46 SUPA-School of Physics and Astronomy, University of Edinburgh, Edinburgh, UK; 47 INFN Laboratori Nazionali di Frascati, Frascati, Italy; 48 Fakultät für Mathematik und Physik, Albert-Ludwigs-Universität, Freiburg, Germany; 49 Section de Physique, Université de Genève, Geneva, Switzerland; 50 INFN Sezione di Genova, Genoa, Italy; Dipartimento di Fisica, Università di Genova, Genoa, Italy; 51 E. Andronikashvili Institute of Physics, Iv. Javakhishvili Tbilisi State University, Tbilisi, Georgia; High Energy Physics Institute, Tbilisi State University, Tbilisi, Georgia; 52 II Physikalisches Institut, Justus-Liebig-Universität Giessen, Giessen, Germany; 53 SUPA-School of Physics and Astronomy, University of Glasgow, Glasgow, UK; 54 II Physikalisches Institut, Georg-August-Universität, Göttingen, Germany; 55 Laboratoire de Physique Subatomique et de Cosmologie, Université Grenoble-Alpes, CNRS/IN2P3, Grenoble, France; 56 Department of Physics, Hampton University, Hampton, VA USA; 57 Laboratory for Particle Physics and Cosmology, Harvard University, Cambridge, MA USA; 58 Kirchhoff-Institut für Physik, Ruprecht-Karls-Universität Heidelberg, Heidelberg, Germany; Physikalisches Institut, Ruprecht-Karls-Universität Heidelberg, Heidelberg, Germany; ZITI Institut für technische Informatik, Ruprecht-Karls-Universität Heidelberg, Mannheim, Germany; 59 Faculty of Applied Information Science, Hiroshima Institute of Technology, Hiroshima, Japan; 60 Department of Physics, Indiana University, Bloomington, IN USA; 61 Institut für Astro- und Teilchenphysik, Leopold-Franzens-Universität, Innsbruck, Austria; 62 University of Iowa, Iowa City, IA USA; 63 Department of Physics and Astronomy, Iowa State University, Ames, IA USA; 64 Joint Institute for Nuclear Research, JINR Dubna, Dubna, Russia; 65 KEK, High Energy Accelerator Research Organization, Tsukuba, Japan; 66 Graduate School of Science, Kobe University, Kobe, Japan; 67 Faculty of Science, Kyoto University, Kyoto, Japan; 68 Kyoto University of Education, Kyoto, Japan; 69 Department of Physics, Kyushu University, Fukuoka, Japan; 70 Instituto de Física La Plata, Universidad Nacional de La Plata and CONICET, La Plata, Argentina; 71 Physics Department, Lancaster University, Lancaster, UK; 72 INFN Sezione di Lecce, Lecce, Italy; Dipartimento di Matematica e Fisica, Università del Salento, Lecce, Italy; 73 Oliver Lodge Laboratory, University of Liverpool, Liverpool, UK; 74 Department of Physics, Jožef Stefan Institute and University of Ljubljana, Ljubljana, Slovenia; 75 School of Physics and Astronomy, Queen Mary University of London, London, UK; 76 Department of Physics, Royal Holloway University of London, Surrey, UK; 77 Department of Physics and Astronomy, University College London, London, UK; 78 Louisiana Tech University, Ruston, LA USA; 79 Laboratoire de Physique Nucléaire et de Hautes Energies, UPMC and Université Paris-Diderot and CNRS/IN2P3, Paris, France; 80 Fysiska institutionen, Lunds universitet, Lund, Sweden; 81 Departamento de Fisica Teorica C-15, Universidad Autonoma de Madrid, Madrid, Spain; 82 Institut für Physik, Universität Mainz, Mainz, Germany; 83 School of Physics and Astronomy, University of Manchester, Manchester, UK; 84 CPPM, Aix-Marseille Université and CNRS/IN2P3, Marseille, France; 85 Department of Physics, University of Massachusetts, Amherst, MA USA; 86 Department of Physics, McGill University, Montreal, QC Canada; 87 School of Physics, University of Melbourne, Parkville, VIC Australia; 88 Department of Physics, The University of Michigan, Ann Arbor, MI USA; 89 Department of Physics and Astronomy, Michigan State University, East Lansing, MI USA; 90 INFN Sezione di Milano, Milan, Italy; Dipartimento di Fisica, Università di Milano, Milan, Italy; 91 B.I. Stepanov Institute of Physics, National Academy of Sciences of Belarus, Minsk, Republic of Belarus; 92 National Scientific and Educational Centre for Particle and High Energy Physics, Minsk, Republic of Belarus; 93 Department of Physics, Massachusetts Institute of Technology, Cambridge, MA USA; 94 Group of Particle Physics, University of Montreal, Montreal, QC Canada; 95 P.N. Lebedev Institute of Physics, Academy of Sciences, Moscow, Russia; 96 Institute for Theoretical and Experimental Physics (ITEP), Moscow, Russia; 97 Moscow Engineering and Physics Institute (MEPhI), Moscow, Russia; 98 D.V.Skobeltsyn Institute of Nuclear Physics, M.V.Lomonosov Moscow State University, Moscow, Russia; 99 Fakultät für Physik, Ludwig-Maximilians-Universität München, Munich, Germany; 100 Max-Planck-Institut für Physik (Werner-Heisenberg-Institut), Munich, Germany; 101 Nagasaki Institute of Applied Science, Nagasaki, Japan; 102 Graduate School of Science and Kobayashi-Maskawa Institute, Nagoya University, Nagoya, Japan; 103 INFN Sezione di Napoli, Naples, Italy; Dipartimento di Fisica, Università di Napoli, Naples, Italy; 104 Department of Physics and Astronomy, University of New Mexico, Albuquerque, NM USA; 105 Institute for Mathematics, Astrophysics and Particle Physics, Radboud University Nijmegen/Nikhef, Nijmegen, The Netherlands; 106 Nikhef National Institute for Subatomic Physics and University of Amsterdam, Amsterdam, The Netherlands; 107 Department of Physics, Northern Illinois University, DeKalb, IL USA; 108 Budker Institute of Nuclear Physics, SB RAS, Novosibirsk, Russia; 109 Department of Physics, New York University, New York, NY USA; 110 Ohio State University, Columbus, OH USA; 111 Faculty of Science, Okayama University, Okayama, Japan; 112 Homer L. Dodge Department of Physics and Astronomy, University of Oklahoma, Norman, OK USA; 113 Department of Physics, Oklahoma State University, Stillwater, OK USA; 114 Palacký University, RCPTM, Olomouc, Czech Republic; 115 Center for High Energy Physics, University of Oregon, Eugene, OR USA; 116 LAL, Université Paris-Sud and CNRS/IN2P3, Orsay, France; 117 Graduate School of Science, Osaka University, Osaka, Japan; 118 Department of Physics, University of Oslo, Oslo, Norway; 119 Department of Physics, Oxford University, Oxford, UK; 120 INFN Sezione di Pavia, Pavia, Italy; Dipartimento di Fisica, Università di Pavia, Pavia, Italy; 121 Department of Physics, University of Pennsylvania, Philadelphia, PA USA; 122 Petersburg Nuclear Physics Institute, Gatchina, Russia; 123 INFN Sezione di Pisa, Pisa, Italy; Dipartimento di Fisica E. Fermi, Università di Pisa, Pisa, Italy; 124 Department of Physics and Astronomy, University of Pittsburgh, Pittsburgh, PA USA; 125 Laboratorio de Instrumentacao e Fisica Experimental de Particulas-LIP, Lisbon, Portugal; Faculdade de Ciências, Universidade de Lisboa, Lisbon, Portugal; Department of Physics, University of Coimbra, Coimbra, Portugal; Centro de Física Nuclear da Universidade de Lisboa, Lisbon, Portugal; Departamento de Fisica, Universidade do Minho, Braga, Portugal; Departamento de Fisica Teorica y del Cosmos and CAFPE, Universidad de Granada, Granada, Spain; Dep Fisica and CEFITEC of Faculdade de Ciencias e Tecnologia, Universidade Nova de Lisboa, Caparica, Portugal; 126 Institute of Physics, Academy of Sciences of the Czech Republic, Prague, Czech Republic; 127 Czech Technical University in Prague, Prague, Czech Republic; 128 Faculty of Mathematics and Physics, Charles University in Prague, Prague, Czech Republic; 129 State Research Center Institute for High Energy Physics, Protvino, Russia; 130 Particle Physics Department, Rutherford Appleton Laboratory, Didcot, UK; 131 Physics Department, University of Regina, Regina, SK Canada; 132 Ritsumeikan University, Kusatsu, Shiga Japan; 133 INFN Sezione di Roma, Rome, Italy; Dipartimento di Fisica, Sapienza Università di Roma, Rome, Italy; 134 INFN Sezione di Roma Tor Vergata, Rome, Italy; Dipartimento di Fisica, Università di Roma Tor Vergata, Rome, Italy; 135 INFN Sezione di Roma Tre, Rome, Italy; Dipartimento di Matematica e Fisica, Università Roma Tre, Rome, Italy; 136 Faculté des Sciences Ain Chock, Réseau Universitaire de Physique des Hautes Energies-Université Hassan II, Casablanca, Morocco; Centre National de l’Energie des Sciences Techniques Nucleaires, Rabat, Morocco; Faculté des Sciences Semlalia, Université Cadi Ayyad, LPHEA-Marrakech, Marrakech, Morocco; Faculté des Sciences, Université Mohamed Premier and LPTPM, Oujda, Morocco; Faculté des Sciences, Université Mohammed V-Agdal, Rabat, Morocco; 137 DSM/IRFU (Institut de Recherches sur les Lois Fondamentales de l’Univers), CEA Saclay (Commissariat à l’Energie Atomique et aux Energies Alternatives), Gif-sur-Yvette, France; 138 Santa Cruz Institute for Particle Physics, University of California Santa Cruz, Santa Cruz, CA USA; 139 Department of Physics, University of Washington, Seattle, WA USA; 140 Department of Physics and Astronomy, University of Sheffield, Sheffield, UK; 141 Department of Physics, Shinshu University, Nagano, Japan; 142 Fachbereich Physik, Universität Siegen, Siegen, Germany; 143 Department of Physics, Simon Fraser University, Burnaby, BC Canada; 144 SLAC National Accelerator Laboratory, Stanford, CA USA; 145 Faculty of Mathematics, Physics and Informatics, Comenius University, Bratislava, Slovak Republic; Department of Subnuclear Physics, Institute of Experimental Physics of the Slovak Academy of Sciences, Kosice, Slovak Republic; 146 Department of Physics, University of Cape Town, Cape Town, South Africa; Department of Physics, University of Johannesburg, Johannesburg, South Africa; School of Physics, University of the Witwatersrand, Johannesburg, South Africa; 147 Department of Physics, Stockholm University, Stockholm, Sweden; The Oskar Klein Centre, Stockholm, Sweden; 148 Physics Department, Royal Institute of Technology, Stockholm, Sweden; 149 Departments of Physics and Astronomy and Chemistry, Stony Brook University, Stony Brook, NY USA; 150 Department of Physics and Astronomy, University of Sussex, Brighton, UK; 151 School of Physics, University of Sydney, Sydney, Australia; 152 Institute of Physics, Academia Sinica, Taipei, Taiwan; 153 Department of Physics, Technion: Israel Institute of Technology, Haifa, Israel; 154 Raymond and Beverly Sackler School of Physics and Astronomy, Tel Aviv University, Tel Aviv, Israel; 155 Department of Physics, Aristotle University of Thessaloniki, Thessaloniki, Greece; 156 International Center for Elementary Particle Physics and Department of Physics, The University of Tokyo, Tokyo, Japan; 157 Graduate School of Science and Technology, Tokyo Metropolitan University, Tokyo, Japan; 158 Department of Physics, Tokyo Institute of Technology, Tokyo, Japan; 159 Department of Physics, University of Toronto, Toronto, ON Canada; 160 TRIUMF, Vancouver, BC, Canada; Department of Physics and Astronomy, York University, Toronto, ON Canada; 161 Faculty of Pure and Applied Sciences, University of Tsukuba, Tsukuba, Japan; 162 Department of Physics and Astronomy, Tufts University, Medford, MA USA; 163 Centro de Investigaciones, Universidad Antonio Narino, Bogotá, Colombia; 164 Department of Physics and Astronomy, University of California Irvine, Irvine, CA USA; 165 INFN Gruppo Collegato di Udine, Sezione di Trieste, Udine, Italy; ICTP, Trieste, Italy; Dipartimento di Chimica, Fisica e Ambiente, Università di Udine, Udine, Italy; 166 Department of Physics, University of Illinois, Urbana, IL USA; 167 Department of Physics and Astronomy, University of Uppsala, Uppsala, Sweden; 168 Instituto de Física Corpuscular (IFIC) and Departamento de Física Atómica, Molecular y Nuclear and Departamento de Ingeniería Electrónica and Instituto de Microelectrónica de Barcelona (IMB-CNM), University of Valencia and CSIC, Valencia, Spain; 169 Department of Physics, University of British Columbia, Vancouver, BC Canada; 170 Department of Physics and Astronomy, University of Victoria, Victoria, BC Canada; 171 Department of Physics, University of Warwick, Coventry, UK; 172 Waseda University, Tokyo, Japan; 173 Department of Particle Physics, The Weizmann Institute of Science, Rehovot, Israel; 174 Department of Physics, University of Wisconsin, Madison, WI USA; 175 Fakultät für Physik und Astronomie, Julius-Maximilians-Universität, Würzburg, Germany; 176 Fachbereich C Physik, Bergische Universität Wuppertal, Wuppertal, Germany; 177 Department of Physics, Yale University, New Haven, CT USA; 178 Yerevan Physics Institute, Yerevan, Armenia; 179 Centre de Calcul de l’Institut National de Physique Nucléaire et de Physique des Particules (IN2P3), Villeurbanne, France

## Abstract

The inclusive top quark pair ($t\overline{t}$) production cross-section ${\sigma _{t\overline{t}}}$ has been measured in proton–proton collisions at ${\sqrt{s}=7~\mathrm{TeV}}$ and ${\sqrt{s}=8~\mathrm{TeV}}$ with the ATLAS experiment at the LHC, using $t\overline{t}$ events with an opposite-charge $e\mu$ pair in the final state. The measurement was performed with the 2011 7 TeV dataset corresponding to an integrated luminosity of 4.6 ${\mathrm{fb}^{-1}}$ and the 2012 8 TeV dataset of 20.3 ${\mathrm{fb}^{-1}}$. The numbers of events with exactly one and exactly two $b$-tagged jets were counted and used to simultaneously determine ${\sigma _{t\overline{t}}}$ and the efficiency to reconstruct and $b$-tag a jet from a top quark decay, thereby minimising the associated systematic uncertainties. The cross-section was measured to be:

$$\begin{aligned} {\sigma _{t\overline{t}}}&= 182.9\pm 3.1\pm 4.2\pm 3.6\pm 3.3~\mathrm{pb}\ ({\sqrt{s}=7~\mathrm{TeV}})\quad \mathrm{and} \\ {\sigma _{t\overline{t}}}&= 242.4\pm 1.7\pm 5.5\pm 7.5\pm 4.2~\mathrm{pb}\ ({\sqrt{s}=8~\mathrm{TeV}}), \end{aligned}$$

where the four uncertainties arise from data statistics, experimental and theoretical systematic effects, knowledge of the integrated luminosity and of the LHC beam energy. The results are consistent with recent theoretical QCD calculations at next-to-next-to-leading order. Fiducial measurements corresponding to the experimental acceptance of the leptons are also reported, together with the ratio of cross-sections measured at the two centre-of-mass energies. The inclusive cross-section results were used to determine the top quark pole mass via the dependence of the theoretically predicted cross-section on ${m}_{t}^\mathrm{pole}$ giving a result of ${m}_{t}^\mathrm{pole}$ $=172.9^{+2.5}_{-2.6}$ GeV. By looking for an excess of $t\overline{t}$ production with respect to the QCD prediction, the results were also used to place limits on the pair-production of supersymmetric top squarks ${\tilde{t}_{1}}$ with masses close to the top quark mass, decaying via ${\tilde{t}_{1}}\rightarrow t{\tilde{\chi }_{1}^{0}}$ to predominantly right-handed top quarks and a light neutralino $\tilde{\chi }_{1}^{0}$, the lightest supersymmetric particle. Top squarks with masses between the top quark mass and 177 GeV are excluded at the 95 % confidence level.

## Introduction

The top quark is the heaviest known fundamental particle, with a mass (${m}_{t}$) that is much larger than any of the other quarks, and close to the scale of electroweak symmetry breaking. The study of its production and decay properties forms a core part of the ATLAS physics programme at the CERN Large Hadron Collider (LHC). At the LHC, top quarks are primarily produced in quark–antiquark pairs ($t\overline{t}$), and the precise prediction of the corresponding inclusive cross-section (${\sigma _{t\overline{t}}}$) is a substantial challenge for quantum chromodynamics (QCD) calculational techniques. Precise measurements of ${\sigma _{t\overline{t}}}$ are sensitive to the gluon parton distribution function (PDF), the top quark mass, and potential enhancements of the cross-section due to physics beyond the Standard Model.

Within the Standard Model (SM), the top quark decays almost exclusively to a $W$ boson and a $b$ quark, so the final-state topologies in $t\overline{t}$ production are governed by the decay modes of the two $W$ bosons. This paper describes a measurement in the dileptonic $e\mu$ channel, $t\overline{t}\rightarrow W^{+}bW^{-}\bar{b}\rightarrow {e}^{\pm }\mu ^{\mp }{\nu }{{\overline{\nu }}}{{b\overline{b}}}$, selecting events with an $e\mu$ pair with opposite-sign electric charges,^1^ and one or two hadronic jets from the $b$ quarks. Jets originating from $b$ quarks were identified (‘tagged’) using a $b$-tagging algorithm exploiting the long lifetime, high decay multiplicity, hard fragmentation and high mass of $B$ hadrons. The rates of events with an $e\mu$ pair and one or two tagged $b$-jets were used to measure simultaneously the $t\overline{t}$ production cross-section and the combined probability to reconstruct and $b$-tag a jet from a top quark decay. Events with electrons or muons produced via leptonic $\tau$ decays $t\rightarrow Wb\rightarrow \tau \nu b\rightarrow e/\mu \nu \nu \nu b$, were included as part of the $t\overline{t}$ signal.

The main background is $Wt$, the associated production of a $W$ boson and a single top quark. Other background contributions arise from $Z\rightarrow \tau \tau \rightarrow e\mu$+jets ($+4\nu$) production, diboson+jets production and events where at least one reconstructed lepton does not arise from a $W$ or $Z$ boson decay.

Theoretical predictions for ${\sigma _{t\overline{t}}}$ are described in Sect. 2, followed by the data and Monte Carlo (MC) simulation samples in Sect. 3, the object and event selection in Sect. 4, and the extraction of the $t\overline{t}$ cross-section in Sect. 5. Systematic uncertainties are discussed in Sect. 6, the results, including fiducial cross-section measurements, the extraction of the top quark mass from the measured cross-section and a limit on the production of supersymmetric top squarks, are given in Sect. 7, and conclusions are drawn in Sect. 8.

## Theoretical cross-section predictions

Calculations of ${\sigma _{t\overline{t}}}$ for hadron collisions are now available at full next-to-next-to-leading-order (NNLO) accuracy in the strong coupling constant $\alpha _\mathrm{s}$, including the resummation of next-to-next-to-leading logarithmic (NNLL) soft gluon terms [1–6]. At a centre-of-mass energy of ${\sqrt{s}\,{=}\,7~\mathrm{TeV}}$ and assuming ${m}_{t}$ $=172.5$ GeV, these calculations give a prediction of $177.3\pm 9.0\,^{+4.6}_{-6.0}$ pb, where the first uncertainty is due to PDF and $\alpha _\mathrm{s}$ uncertainties, and the second to QCD scale uncertainties. The corresponding prediction at ${\sqrt{s}=8~\mathrm{TeV}}$ is $252.9\pm 11.7\,^{+6.4}_{-8.6}$ pb. These values were calculated using the top++ 2.0 program [7]. The PDF and $\alpha _\mathrm{s}$ uncertainties were calculated using the PDF4LHC prescription [8] with the MSTW2008 68 % CL NNLO [9, 10], CT10 NNLO [11, 12] and NNPDF2.3 5f FFN [13] PDF sets, and added in quadrature to the QCD scale uncertainty. The latter was obtained from the envelope of predictions with the renormalisation and factorisation scales varied independently by factors of two up and down from their default values of ${m}_{t}$, whilst never letting them differ by more than a factor of two. The ratio of cross-sections at $\sqrt{s}=8~$ TeV and $\sqrt{s}=7~$ TeV is predicted to be $1.430\pm 0.013$ (PDF+$\alpha _\mathrm{s}$) $\pm 0.001$ (QCD scale). The total relative uncertainty is only 0.9 %, as the cross-section uncertainties at the two centre-of-mass energies are highly correlated.

The NNLO+NNLL cross-section values are about 3 % larger than the exact NNLO predictions, as implemented in Hathor 1.5 [14]. For comparison, the corresponding next-to-leading-order (NLO) predictions, also calculated using top++ 2.0 with the same set of PDFs, are $157\pm 12\pm 24$ pb at $\sqrt{s}=7~$ TeV and $225\pm 16\pm 29$ pb at $\sqrt{s}=8~$ TeV, where again the first quoted uncertainties are due to PDF and $\alpha _\mathrm{s}$ uncertainties, and the second to QCD scale uncertainties. The total uncertainties of the NLO predictions are approximately 15 %, about three times larger than the NNLO+NNLL calculation uncertainties quoted above.

## Data and simulated samples

The ATLAS detector [15] at the LHC covers nearly the entire solid angle around the collision point, and consists of an inner tracking detector surrounded by a thin superconducting solenoid magnet producing a 2 T axial magnetic field, electromagnetic and hadronic calorimeters, and an external muon spectrometer incorporating three large toroid magnet assemblies. The inner detector consists of a high-granularity silicon pixel detector and a silicon microstrip tracker, together providing precision tracking in the pseudorapidity^2^ range $|\eta |<2.5$, complemented by a transition radiation tracker providing tracking and electron identification information for $|\eta |<2.0$. A lead/liquid-argon (LAr) electromagnetic calorimeter covers the region $|\eta |<3.2$, and hadronic calorimetry is provided by steel/scintillator tile calorimeters for $|\eta |<1.7$ and copper/LAr hadronic endcap calorimeters. The forward region is covered by additional LAr calorimeters with copper and tungsten absorbers. The muon spectrometer consists of precision tracking chambers covering the region $|\eta |<2.7$, and separate trigger chambers covering $|\eta |<2.4$. A three-level trigger system, using custom hardware followed by two software-based levels, is used to reduce the event rate to about 400 Hz for offline storage.

The analysis was performed on the ATLAS 2011–2012 proton–proton collision data sample, corresponding to integrated luminosities of 4.6 ${\mathrm{fb}^{-1}}$ at $\sqrt{s}=7~$ TeV and 20.3 ${\mathrm{fb}^{-1}}$ at $\sqrt{s}=8~$ TeV after the application of detector status and data quality requirements. Events were required to pass either a single-electron or single-muon trigger, with thresholds chosen in each case such that the efficiency plateau is reached for leptons with $p_\mathrm{T}>25$ GeV passing offline selections. Due to the high instantaneous luminosities achieved by the LHC, each triggered event also includes the signals from on average about 9 ($\sqrt{s}=7~$ TeV) or 20 ($\sqrt{s}=8~$ TeV) additional inelastic $pp$ collisions in the same bunch crossing (known as pileup).

Monte Carlo simulated event samples were used to develop the analysis, to compare to the data and to evaluate signal and background efficiencies and uncertainties. Samples were processed either through the full ATLAS detector simulation [16] based on GEANT4 [17], or through a faster simulation making use of parameterised showers in the calorimeters [18]. Additional simulated $pp$ collisions generated either with Pythia6 [19] (for ${\sqrt{s}\,{=}\,7~\mathrm{TeV}}$ simulation) or Pythia8 [20] (for ${\sqrt{s}=8~\mathrm{TeV}}$) were overlaid to simulate the effects of both in- and out-of-time pileup, from additional $pp$ collisions in the same and nearby bunch crossings. All simulated events were then processed using the same reconstruction algorithms and analysis chain as the data. Small corrections were applied to lepton trigger and selection efficiencies to better model the performance seen in data, as discussed further in Sect. 6.

The baseline $t\overline{t}$ full simulation sample was produced using the NLO matrix element generator Powheg [21–23] interfaced to Pythia6 [19] with the Perugia 2011C tune (P2011C) [24] for parton shower, fragmentation and underlying event modelling, and CT10 PDFs [11], and included all $t\overline{t}$ final states involving at least one lepton. The $W\rightarrow \ell \nu$ branching ratio was set to the SM expectation of $0.1082$ [25], and ${m}_{t}$ was set to 172.5 GeV. Alternative $t\overline{t}$ samples were produced with the NLO generator MC@NLO [26, 27] interfaced to Herwig [28] with Jimmy [29] for the underlying event modelling, with the ATLAS AUET2 [30] tune and CT10 PDFs; and with the leading-order (LO) multileg generator Alpgen [31] interfaced to either Pythia6 or Herwig and Jimmy, with the CTEQ6L1 PDFs [32]. These samples were all normalised to the NNLO+NNLL cross-section predictions given in Sect. 2 when comparing simulation with data.

Backgrounds were classified into two types: those with two real prompt leptons from $W$ or $Z$ boson decays (including those produced via leptonic $\tau$ decays), and those where at least one of the reconstructed lepton candidates is misidentified, i.e. a non-prompt lepton from the decay of a bottom or charm hadron, an electron from a photon conversion, hadronic jet activity misidentified as an electron, or a muon produced from an in-flight decay of a pion or kaon. The first category with two prompt leptons includes $Wt$ single top production, modelled using Powheg + Pythia6 [33] with the CT10 PDFs and the P2011C tune; $Z\rightarrow \tau \tau$+jets modelled using Alpgen + Herwig + Jimmy ($\sqrt{s}=7~$ TeV) or Alpgen + Pythia6 including LO matrix elements for $Z{b\overline{b}}$ production, with CTEQ6L1 PDFs; and diboson ($WW$, $WZ$, $ZZ$) production in association with jets, modelled using Alpgen + Herwig + Jimmy. The $Wt$ background was normalised to approximate NNLO cross-sections of $15.7\pm 1.2$ pb at ${\sqrt{s}\,{=}\,7~\mathrm{TeV}}$ and $22.4\pm 1.5$ pb at ${\sqrt{s}=8~\mathrm{TeV}}$, determined as in Ref. [34]. The inclusive $Z$ cross-sections were set to the NNLO predictions from FEWZ [35], but the normalisation of $Z\rightarrow \tau \tau \rightarrow e\mu 4\nu$ backgrounds with $b$-tagged jets were determined from data as described in Sect. 5.1. The diboson background was normalised to the NLO QCD inclusive cross-section predictions calculated with MCFM [36]. Production of $t\overline{t}$ in association with a $W$ or $Z$ boson, which contributes to the sample with same-sign leptons, was simulated with Madgraph [37] interfaced to Pythia with CTEQ6L1 PDFs, and normalised to NLO cross-section predictions [38, 39].

Backgrounds with one real and one misidentified lepton include $t\overline{t}$ events with one hadronically decaying $W$; $W$+jets production, modelled as for $Z$+jets; $W\gamma$+jets, modelled with Sherpa [40] with CT10 PDFs; and $t$-channel single top production, modelled using AcerMC [41] interfaced to Pythia6 with CTEQ6L1 PDFs. Other backgrounds, including processes with two misidentified leptons, are negligible after the event selections used in this analysis.

## Object and event selection

The analysis makes use of reconstructed electrons, muons and $b$-tagged jets. Electron candidates were reconstructed from an isolated electromagnetic calorimeter energy deposit matched to an inner detector track and passing tight identification requirements [42], with transverse energy $E_\mathrm{T}>25$ GeV and pseudorapidity $|\eta |<2.47$. Electron candidates within the transition region between the barrel and endcap electromagnetic calorimeters, $1.37<|\eta |<1.52$, were removed. Isolation requirements were used to reduce background from non-prompt electrons. The calorimeter transverse energy within a cone of size $\varDelta R=0.2$ and the scalar sum of track $p_\mathrm{T}$ within a cone of size $\varDelta R=0.3$, in each case excluding the contribution from the electron itself, were each required to be smaller than $E_\mathrm{T}$ and $\eta$-dependent thresholds calibrated to separately give nominal selection efficiencies of 98 % for prompt electrons from $Z\rightarrow ee$ decays.

Muon candidates were reconstructed by combining matching tracks reconstructed in both the inner detector and muon spectrometer [43], and were required to satisfy $p_\mathrm{T}>25$ GeV and $|\eta |<2.5$. In the $\sqrt{s}=7~$ TeV dataset, the calorimeter transverse energy within a cone of size $\varDelta R=0.2$, excluding the energy deposited by the muon, was required to be less than 4 GeV, and the scalar sum of track $p_\mathrm{T}$ within a cone of size $\varDelta R=0.3$, excluding the muon track, was required to be less than 2.5 GeV. In the $\sqrt{s}=8~$ TeV dataset, these isolation requirements were replaced by a cut $I<0.05$, where $I$ is the ratio of the sum of track $p_\mathrm{T}$ in a variable-sized cone of radius $\varDelta R=10~\mathrm{~{\hbox {GeV}}}/p_\mathrm{T}^\mu$ to the transverse momentum $p_\mathrm{T}^\mu$ of the muon [44]. Both sets of isolation requirements have efficiencies of about 97 % for prompt muons from $Z\rightarrow \mu \mu$ decays.

Jets were reconstructed using the anti-$k_t$ algorithm [45, 46] with radius parameter $R=0.4$, starting from calorimeter energy clusters calibrated at the electromagnetic energy scale for the $\sqrt{s}=7~$ TeV dataset, or using the local cluster weighting method for ${\sqrt{s}=8~\mathrm{TeV}}$ [47]. Jets were calibrated using an energy- and $\eta$-dependent simulation-based calibration scheme, with in-situ corrections based on data, and were required to satisfy $p_\mathrm{T}>25$ GeV and $|\eta |<2.5$. To suppress the contribution from low-$p_\mathrm{T}$ jets originating from pileup interactions, a jet vertex fraction requirement was applied: at ${\sqrt{s}\,{=}\,7~\mathrm{TeV}}$ jets were required to have at least 75 % of the scalar sum of the $p_\mathrm{T}$ of tracks associated with the jet coming from tracks associated with the event primary vertex. The latter was defined as the reconstructed vertex with the highest sum of associated track $p_\mathrm{T}^2$. Motivated by the higher pileup background, in the $\sqrt{s}=8~$ TeV dataset this requirement was loosened to 50 %, only applied to jets with $p_\mathrm{T}<50$ GeV and $|\eta |<2.4$, and the effects of pileup on the jet energy calibration were further reduced using the jet-area method as described in Ref. [48]. Finally, to further suppress non-isolated leptons likely to have come from heavy-flavour decays inside jets, electrons and muons within $\varDelta R=0.4$ of selected jets were also discarded.

Jets were $b$-tagged as likely to have originated from $b$ quarks using the MV1 algorithm, a multivariate discriminant making use of track impact parameters and reconstructed secondary vertices [49, 50]. Jets were defined to be $b$-tagged if the MV1 discriminant value was larger than a threshold (working point) corresponding approximately to a 70 % efficiency for tagging $b$-quark jets from top decays in $t\overline{t}$ events, with a rejection factor of about 140 against light-quark and gluon jets, and about five against jets originating from charm quarks.

Events were required to have at least one reconstructed primary vertex with at least five associated tracks, and no jets failing jet quality and timing requirements. Events with muons compatible with cosmic-ray interactions and muons losing substantial fractions of their energy through bremsstrahlung in the detector material were also removed. A preselection requiring exactly one electron and one muon selected as described above was then applied, with at least one of the leptons being matched to an electron or muon object triggering the event. Events with an opposite-sign $e\mu$ pair constituted the main analysis sample, whilst events with a same-sign $e\mu$ pair were used in the estimation of the background from misidentified leptons.

## Extraction of the $t\overline{t}$ cross-section

The $t\overline{t}$ production cross-section ${\sigma _{t\overline{t}}}$ was determined by counting the numbers of opposite-sign $e\mu$ events with exactly one ($N_1$) and exactly two ($N_2$) $b$-tagged jets. No requirements were made on the number of untagged jets; such jets originate from $b$-jets from top decays which were not tagged, and light-quark, charm-quark or gluon jets from QCD radiation. The two event counts can be expressed as:

1 $$\begin{aligned}&\!\!\!N_1= L {{\sigma _{t\overline{t}}}}\, {{\epsilon _{e\mu }}} {2{\epsilon _{b}}} (1-{{{C}_{b}}}{{\epsilon _{b}}}) + {N_1^\mathrm{bkg}}\nonumber \\&\!\!\!N_2= L {{\sigma _{t\overline{t}}}}\, {{\epsilon _{e\mu }}} {{{C}_{b}}} {{\epsilon _{b}}}^{2} + {N_2^\mathrm{bkg}}\end{aligned}$$

where $L$ is the integrated luminosity of the sample, ${\epsilon _{e\mu }}$ is the efficiency for a $t\overline{t}$ event to pass the opposite-sign $e\mu$ preselection and ${{C}_{b}}$ is a tagging correlation coefficient close to unity. The combined probability for a jet from the quark $q$ in the $t\rightarrow Wq$ decay to fall within the acceptance of the detector, be reconstructed as a jet with transverse momentum above the selection threshold, and be tagged as a $b$-jet, is denoted by ${\epsilon _{b}}$. Although this quark is almost always a $b$ quark, ${\epsilon _{b}}$ thus also accounts for the approximately $0.2~\%$ of top quarks that decay to $Ws$ or $Wd$ rather than $Wb$, slightly reducing the effective $b$-tagging efficiency. Furthermore, the value of ${\epsilon _{b}}$ is slightly increased by the small contributions to $N_1$ and $N_2$ from mistagged light-quark, charm-quark or gluon jets from radiation in $t\overline{t}$ events, although more than 98 % of the tagged jets are expected to contain particles from $B$-hadron decays in both the one and two $b$-tag samples.

**Table 1:** Observed numbers of opposite-sign $e\mu$ events with one and two $b$-tagged jets ($N_1$ and $N_2$) for each data sample, together with the estimates of backgrounds and associated total uncertainties described in Sect. 6

Event counts	${\sqrt{s}\,{=}\,7~\mathrm{TeV}}$		${\sqrt{s}=8~\mathrm{TeV}}$	
	$N_1$	$N_2$	$N_1$	$N_2$
Data	3527	2073	21666	11739
$Wt$ single top	$326\pm 36$	$53\pm 14$	$2050\pm 210$	$360\pm 120$
Dibosons	$19\pm 5$	$0.5\pm 0.1$	$120\pm 30$	$3\pm 1$
$Z(\rightarrow \tau \tau \rightarrow e\mu )$+jets	$28\pm 2$	$1.8\pm 0.5$	$210\pm 5$	$7\pm 1$
Misidentified leptons	$27\pm 13$	$15\pm 8$	$210\pm 66$	$95\pm 29$
Total background	$400\pm 40$	$70\pm 16$	$2590\pm 230$	$460\pm 130$

If the decays of the two top quarks and the subsequent reconstruction of the two $b$-tagged jets are completely independent, the probability to tag both $b$-jets ${\epsilon _{bb}}$ is given by ${\epsilon _{bb}}={\epsilon _{b}}^2$. In practice, small correlations are present for both kinematic and instrumental reasons, and these are taken into account via the tagging correlation ${{C}_{b}}$, defined as ${{C}_{b}}={\epsilon _{bb}}/{\epsilon _{b}}^2$ or equivalently ${{C}_{b}}=4 N^{t\overline{t}}_{e\mu } N_2^{t\overline{t}}/(N^{t\overline{t}}_1+2 N^{t\overline{t}}_2)^2$, where $N^{t\overline{t}}_{e\mu }$ is the number of preselected $e\mu$ $t\overline{t}$ events and $N^{t\overline{t}}_1$ and $N^{t\overline{t}}_2$ are the numbers of $t\overline{t}$ events with one and two $b$-tagged jets. Values of ${{C}_{b}}$ greater than one correspond to a positive correlation, where a second jet is more likely to be selected if the first one is already selected, whilst ${{C}_{b}}=1$ corresponds to no correlation. This correlation term also compensates for the effect on ${\epsilon _{b}}$, $N_1$ and $N_2$ of the small number of mistagged charm-quark or gluon jets from radiation in the $t\overline{t}$ events.

**Fig. 1:**
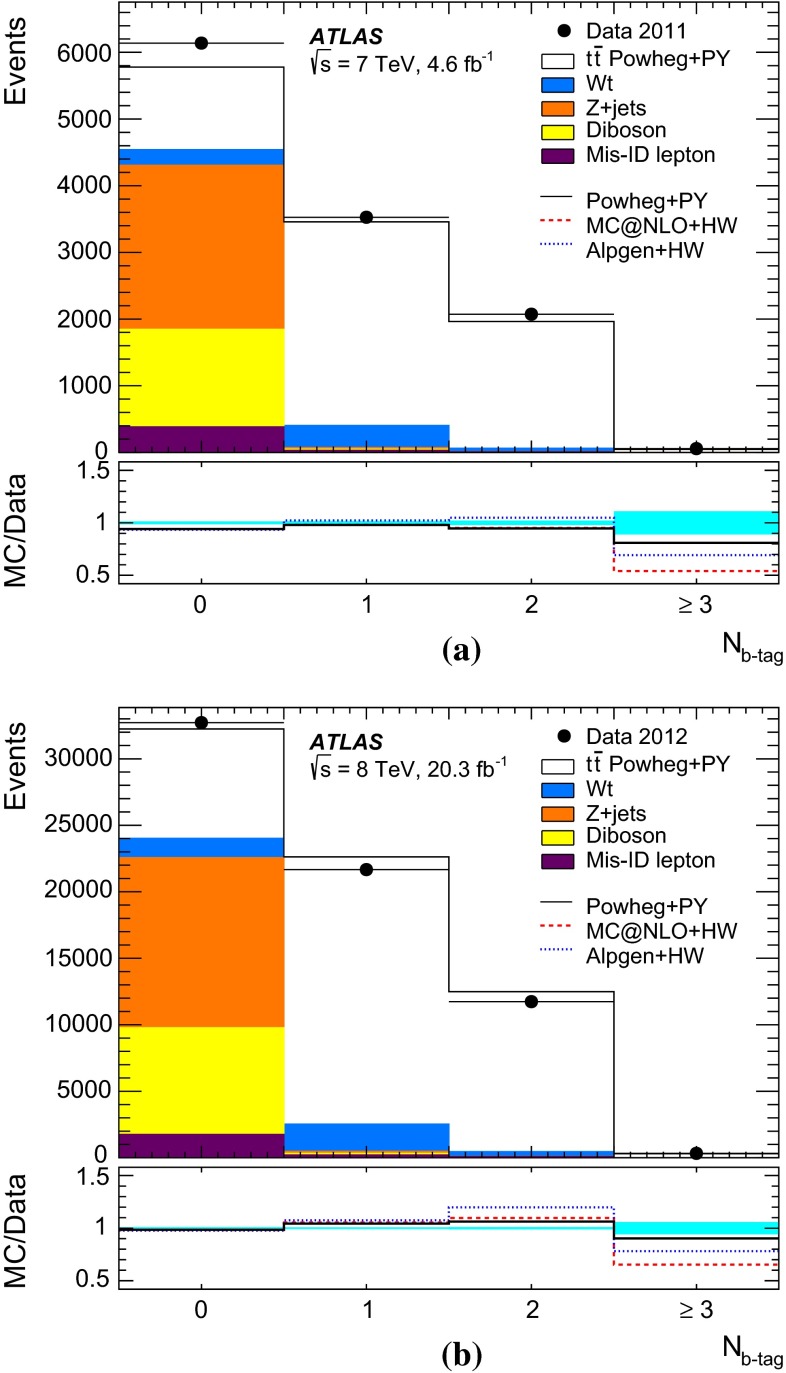
Distributions of the number of $b$-tagged jets in preselected opposite-sign $e\mu$ events in **a** ${\sqrt{s}\,{=}\,7~\mathrm{TeV}}$ and **b** ${\sqrt{s}=8~\mathrm{TeV}}$ data. The data are shown compared to the expectation from simulation, broken down into contributions from $t\overline{t}$, $Wt$ single top, $Z$+jets, dibosons, and events with misidentified electrons or muons, normalised to the same integrated luminosity as the data. The lower parts of the figure show the ratios of simulation to data, using various $t\overline{t}$ signal samples generated with Powheg + Pythia6 (PY), MC@NLO + Herwig (HW) and Alpgen + Herwig, and with the *cyan band* indicating the statistical uncertainty

Background from sources other than $t\overline{t}\rightarrow e\mu \nu {\overline{\nu }}{b\overline{b}}$ also contributes to the event counts $N_1$ and $N_2$, and is given by the terms ${N_1^\mathrm{bkg}}$ and ${N_2^\mathrm{bkg}}$. The preselection efficiency ${\epsilon _{e\mu }}$ and tagging correlation ${{C}_{b}}$ were taken from $t\overline{t}$ event simulation, and the background contributions ${N_1^\mathrm{bkg}}$ and ${N_2^\mathrm{bkg}}$ were estimated using a combination of simulation- and data-based methods, allowing the two equations in Eq. (1) to be solved numerically yielding ${\sigma _{t\overline{t}}}$ and ${\epsilon _{b}}$.

A total of 11796 events passed the $e\mu$ opposite-sign preselection in ${\sqrt{s}\,{=}\,7~\mathrm{TeV}}$ data, and 66453 in ${\sqrt{s}=8~\mathrm{TeV}}$ data. Table 1 shows the number of events with one and two $b$-tagged jets, together with the estimates of non-$t\overline{t}$ background and their systematic uncertainties discussed in detail in Sect. 5.1 below. The samples with one $b$-tagged jet are expected to be about 89 % pure in $t\overline{t}$ events, with the dominant background coming from $Wt$ single top production, and smaller contributions from events with misidentified leptons, $Z$+jets and dibosons. The samples with two $b$-tagged jets are expected to be about 96 % pure in $t\overline{t}$ events, with $Wt$ production again being the dominant background.

**Fig. 2:**
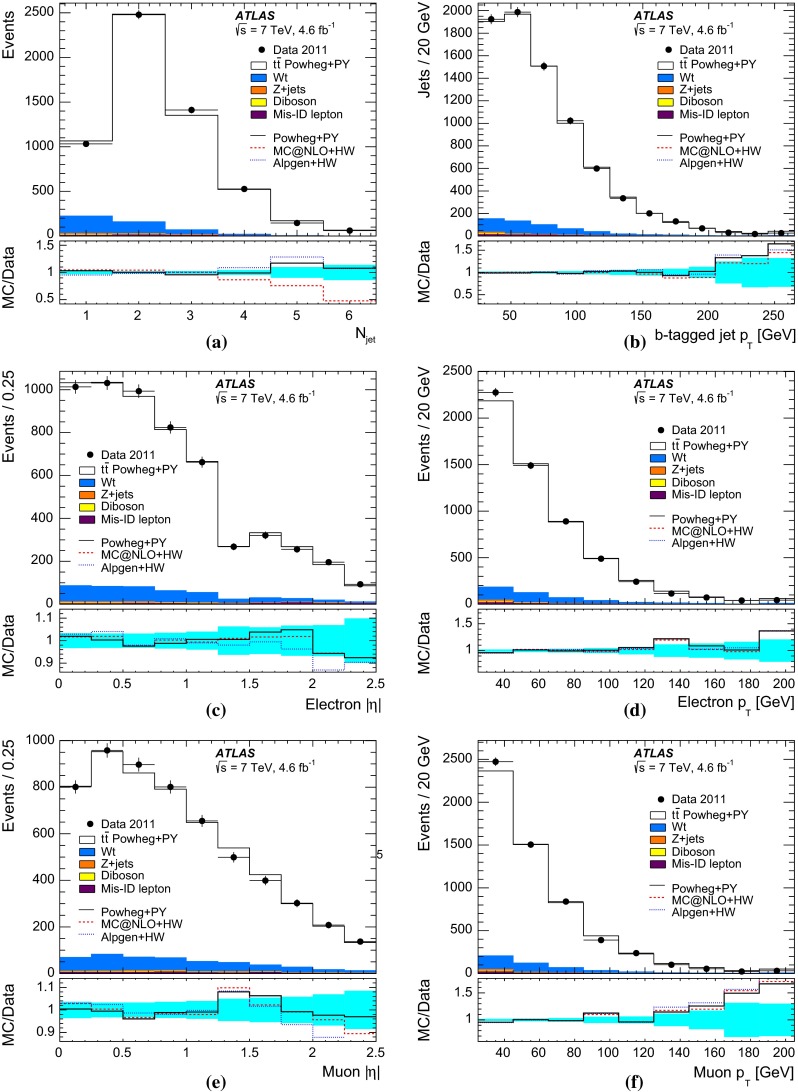
Distributions of **a** the number of jets, **b** the transverse momentum $p_\mathrm{T}$ of the $b$-tagged jets, **c** the $|\eta |$ of the electron, **d** the $p_\mathrm{T}$ of the electron, **e** the $|\eta |$ of the muon and **f** the $p_\mathrm{T}$ of the muon, in events with an opposite-sign $e\mu$ pair and at least one $b$-tagged jet. The ${\sqrt{s}\,{=}\,7~\mathrm{TeV}}$ data are compared to the expectation from simulation, broken down into contributions from $t\overline{t}$, single top, $Z$+jets, dibosons, and events with misidentified electrons or muons, normalised to the same number of entries as the data. The lower parts of the figure show the ratios of simulation to data, using various $t\overline{t}$ signal samples and with the *cyan band* indicating the statistical uncertainty. The last bin includes the overflow

**Fig. 3:**
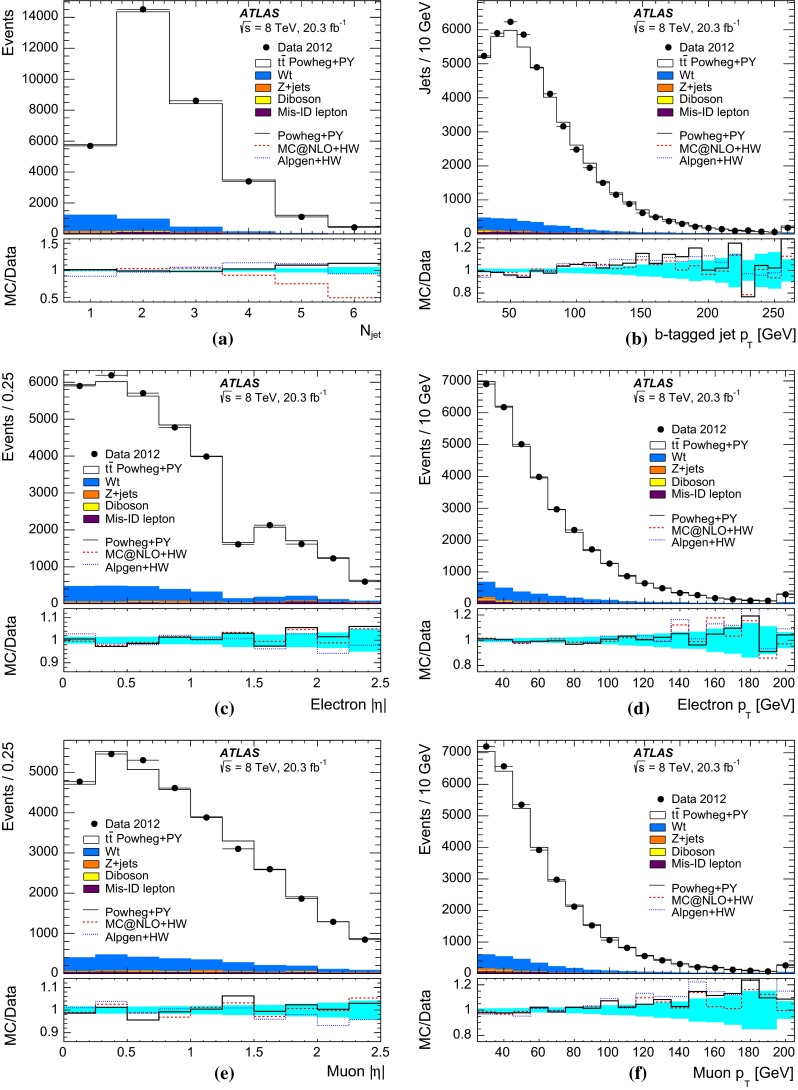
Distributions of **a** the number of jets, **b** the transverse momentum $p_\mathrm{T}$ of the $b$-tagged jets, **c** the $|\eta |$ of the electron, **d** the $p_\mathrm{T}$ of the electron, **e** the $|\eta |$ of the muon and **f** the $p_\mathrm{T}$ of the muon, in events with an opposite-sign $e\mu$ pair and at least one $b$-tagged jet. The ${\sqrt{s}=8~\mathrm{TeV}}$ data are compared to the expectation from simulation, broken down into contributions from $t\overline{t}$, single top, $Z$+jets, dibosons, and events with misidentified electrons or muons, normalised to the same number of entries as the data. The lower parts of the figure show the ratios of simulation to data, using various $t\overline{t}$ signal samples and with the *cyan band* indicating the statistical uncertainty. The last bin includes the overflow

Distributions of the number of $b$-tagged jets in opposite-sign $e\mu$ events are shown in Fig. 1, and compared to the expectations with several $t\overline{t}$ simulation samples. The histogram bins with one and two $b$-tagged jets correspond to the data event counts shown in Table 1. Distributions of the number of jets, the $b$-tagged jet $p_\mathrm{T}$, and the electron and muon $|\eta |$ and $p_\mathrm{T}$ are shown for opposite-sign $e\mu$ events with at least one $b$-tagged jet in Fig. 2 ($\sqrt{s}=7~$ TeV) and Fig. 3 ($\sqrt{s}=8~$ TeV), with the simulation normalised to the same number of entries as the data. The lepton $|\eta |$ distributions reflect the differing acceptances and efficiencies for electrons and muons, in particular the calorimeter transition region at $1.37<|\eta |<1.52$. In general, the agreement between data and simulation is good, within the range of predictions from the different $t\overline{t}$ simulation samples.

The value of ${\sigma _{t\overline{t}}}$ extracted from Eq. (1) is inversely proportional to the assumed value of ${\epsilon _{e\mu }}$, with $(\mathrm{d}{\sigma _{t\overline{t}}}/\mathrm{d}{\epsilon _{e\mu }})$/$({\sigma _{t\overline{t}}}/{\epsilon _{e\mu }})=-1$. Uncertainties on ${\epsilon _{e\mu }}$ therefore translate directly into uncertainties on ${\sigma _{t\overline{t}}}$. The value of ${\epsilon _{e\mu }}$ was determined from simulation to be about 0.8 % for both centre-of-mass energies, and includes the $t\overline{t}\rightarrow e\mu \nu {\overline{\nu }}{b\overline{b}}$ branching ratio of about 3.2 % including $W\rightarrow \tau \rightarrow e/\mu$ decays. Similarly, ${\sigma _{t\overline{t}}}$ is proportional to the value of ${{C}_{b}}$, also determined from simulation, giving a dependence with the opposite sign, $(\mathrm{d}{\sigma _{t\overline{t}}}$ /$\mathrm{d}{{C}_{b}})/({\sigma _{t\overline{t}}}/{{C}_{b}})=1$. The systematic uncertainties on ${\epsilon _{e\mu }}$ and ${{C}_{b}}$ are discussed in Sect. 6.

With the kinematic cuts and $b$-tagging working point chosen for this analysis, the sensitivities of ${\sigma _{t\overline{t}}}$ to knowledge of the backgrounds ${N_1^\mathrm{bkg}}$ and ${N_2^\mathrm{bkg}}$ are given by $(\mathrm{d}{\sigma _{t\overline{t}}}/\mathrm{d}{N_1^\mathrm{bkg}})$/$({\sigma _{t\overline{t}}}/{N_1^\mathrm{bkg}})\,{=}-0.12$ and $(\mathrm{d}{\sigma _{t\overline{t}}}/\mathrm{d}{N_2^\mathrm{bkg}})$/$({\sigma _{t\overline{t}}}/{N_2^\mathrm{bkg}})=-0.004$. The fitted cross-sections are therefore most sensitive to the systematic uncertainties on ${N_1^\mathrm{bkg}}$, whilst for the chosen $b$-tagging working point, the measurements of $N_2$ serve mainly to constrain ${\epsilon _{b}}$. As discussed in Sect. 6.1, consistent results were also obtained at different $b$-tagging efficiency working points that induce greater sensitivity to the background estimate in the two $b$-tag sample.

### Background estimation

The $Wt$ single top and diboson backgrounds were estimated from simulation as discussed in Sect. 3. The $Z$+jets background (with $Z\rightarrow \tau \tau \rightarrow e\mu 4\nu$) at ${\sqrt{s}=8~\mathrm{TeV}}$ was estimated from simulation using Alpgen+Pythia, scaled by the ratios of $Z\rightarrow ee$ or $Z\rightarrow \mu \mu$ accompanied by $b$-tagged jets measured in data and simulation. The ratio was evaluated separately in the one and two $b$-tag event samples. This scaling eliminates uncertainties due to the simulation modelling of jets (especially heavy-flavour jets) produced in association with the $Z$ bosons. The data-to-simulation ratios were measured in events with exactly two opposite-sign electrons or muons passing the selections given in Sect. 4 and one or two $b$-tagged jets, by fitting the dilepton invariant mass distributions in the range 60–120 GeV, accounting for the backgrounds from $t\overline{t}$ production and misidentified leptons. Combining the results from both dilepton channels, the scale factors were determined to be $1.43\pm 0.03$ and $1.13\pm 0.08$ for the one and two $b$-tag backgrounds, after normalising the simulation to the inclusive $Z$ cross-section prediction from FEWZ [35]. The uncertainties include systematic components derived from a comparison of results from the $ee$ and $\mu \mu$ channels, and from studying the variation of scale factors with $Z$ boson $p_\mathrm{T}$. The average $p_\mathrm{T}$ is higher in selected $Z\rightarrow \tau \tau \rightarrow e\mu 4\nu$ events than in $Z\rightarrow ee/\mu \mu$ events due to the momentum lost to the undetected neutrinos from the $\tau$ decays. The same procedure was used for the ${\sqrt{s}\,{=}\,7~\mathrm{TeV}}$ dataset, resulting in scale factors of $1.23\pm 0.07$ (one $b$-tag) and $1.14\pm 0.18$ (two $b$-tags) for the Alpgen + Herwig $Z$+jets simulation, which predicts different numbers of events with heavy-flavour jets than Alpgen + Pythia.

The background from events with one real and one misidentified lepton was estimated using a combination of data and simulation. Simulation studies show that the samples with a same-sign $e\mu$ pair and one or two $b$-tagged jets are dominated by events with misidentified leptons, with rates comparable to those in the opposite-sign sample. The contributions of events with misidentified leptons were therefore estimated using the same-sign event counts in data after subtraction of the estimated prompt same-sign contributions, multiplied by the opposite- to same-sign misidentified-lepton ratios ${{R_j} = N_j^{\mathrm{mis,OS}}}/ {N_j^{\mathrm{mis,SS}}}$ estimated from simulation for events with $j=1$ and 2 $b$-tagged jets. The procedure is illustrated by Table 2, which shows the expected numbers of events with misidentified leptons in opposite- and same-sign samples. The contributions where the electron is misidentified, coming from a photon conversion, the decay of a heavy-flavour hadron or other sources (such as a misidentified hadron within a jet), and where the muon is misidentified, coming either from heavy-flavour decay or other sources (e.g. decay in flight of a pion or kaon) are shown separately. The largest contributions come from photon conversions giving electron candidates, and most of these come from photons radiated from prompt electrons produced from $t\rightarrow Wq\rightarrow e\nu q$ in signal $t\overline{t}\rightarrow e\mu \nu {\overline{\nu }}{b\overline{b}}$ events. Such electrons populate both the opposite- and same-sign samples, and are treated as misidentified-lepton background.

The ratios $R_j$ were estimated from simulation to be $R_1=1.4\pm 0.5$ and $R_2=1.1\pm 0.5$ at ${\sqrt{s}\,{=}\,7~\mathrm{TeV}}$, and $R_1=1.2\pm 0.3$ and $R_2=1.6\pm 0.5$ at ${\sqrt{s}=8~\mathrm{TeV}}$. The uncertainties were derived by considering the range of $R_j$ values for different components of the misidentified-lepton background, including the small contributions from sources other than photon conversions and heavy-flavour decays, which do not significantly populate the same-sign samples. As shown in Table 2, about 25 % of the same-sign events have two prompt leptons, which come mainly from semileptonic $t\overline{t}$ events with an additional leptonically decaying $W$ or $Z$ boson, diboson decays producing two same-sign leptons, and wrong-sign $t\overline{t}\rightarrow e\mu \nu {\overline{\nu }}{b\overline{b}}$ events where the electron charge was misreconstructed. A conservative uncertainty of 50 % was assigned to this background, based on studies of the simulation modelling of electron charge misidentification [42] and uncertainties in the rates of contributing physics processes.

**Fig. 4:**
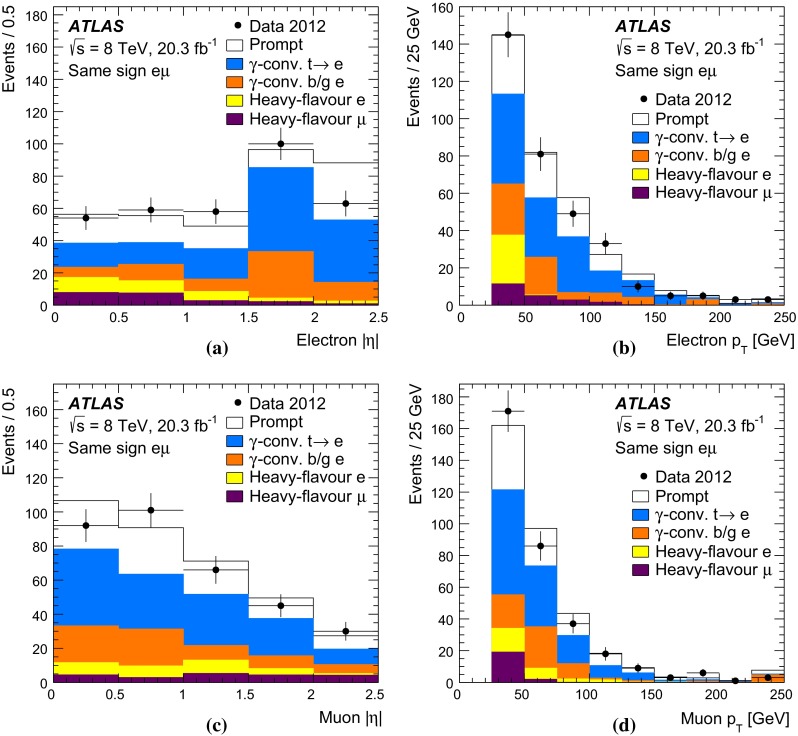
Distributions of electron and muon $|\eta |$ and $p_\mathrm{T}$ in same-sign $e\mu$ events at ${\sqrt{s}=8~\mathrm{TeV}}$ with at least one $b$-tagged jet. The simulation prediction is normalised to the same integrated luminosity as the data, and broken down into contributions where both leptons are prompt, or one is a misidentified lepton from a photon conversion originating from a top quark decay or from background, or from heavy-flavour decay. In the $p_\mathrm{T}$ distributions, the last bin includes the overflows

The simulation modelling of the different components of the misidentified-lepton background was checked by studying kinematic distributions of same-sign events, as illustrated for the $|\eta |$ and $p_\mathrm{T}$ distributions of the leptons in ${\sqrt{s}=8~\mathrm{TeV}}$ data in Fig. 4. The simulation generally models the normalisation and shapes of distributions well in both the one and two $b$-tag event samples. The simulation modelling was further tested in control samples with relaxed electron or muon isolation requirements to enhance the relative contributions of electrons or muons from heavy-flavour decays, and similar levels of agreement were observed.

**Table 2:** Breakdown of estimated misidentified-lepton contributions to the one ($1b$) and two ($2b$) $b$-tag opposite- and same-sign (OS and SS) $e\mu$ event samples at ${\sqrt{s}\,{=}\,7~\mathrm{TeV}}$ and ${\sqrt{s}=8~\mathrm{TeV}}$. The different misidentified-lepton categories are described in the text. For the same-sign samples, the contributions from wrong-sign (where the electron charge sign is misreconstructed) and right-sign prompt lepton events are also shown, and the total expectations are compared to the data. The uncertainties shown are due to the limited size of the simulated samples, and values and uncertainties quoted as ‘0.0’ are smaller than 0.05

Component	${\sqrt{s}\,{=}\,7~\mathrm{TeV}}$				${\sqrt{s}=8~\mathrm{TeV}}$			
	OS $1b$	SS $1b$	OS $2b$	SS $2b$	OS $1b$	SS $1b$	OS $2b$	SS $2b$
$t\rightarrow e\rightarrow \gamma$ conversion $e$	$13.5\pm 0.8$	$11.3\pm 0.8$	$6.1\pm 0.6$	$6.4\pm 0.6$	$97\pm 5$	$93\pm 5$	$67\pm 5$	$44\pm 4$
Background conversion $e$	$7.2\pm 1.3$	$3.3\pm 0.5$	$1.4\pm 0.2$	$0.7\pm 0.2$	$53\pm 11$	$55\pm 12$	$12.8\pm 2.5$	$8.7\pm 1.9$
Heavy-flavour $e$	$2.9\pm 0.4$	$3.8\pm 0.4$	$0.3\pm 0.1$	$0.5\pm 0.1$	$33\pm 4$	$24\pm 3$	$5.6\pm 1.3$	$2.3\pm 0.8$
Other $e$	$2.8\pm 0.7$	$0.0\pm 0.0$	$0.2\pm 0.1$	$0.0\pm 0.0$	$17\pm 7$	$0.5\pm 0.3$	$4.7\pm 1.2$	$0.1\pm 0.1$
Heavy-flavour $\mu$	$3.2\pm 0.4$	$3.0\pm 0.4$	$0.5\pm 0.2$	$0.1\pm 0.1$	$26\pm 6$	$17.9\pm 2.7$	$2.4\pm 0.8$	$2.8\pm 1.0$
Other $\mu$	$0.7\pm 0.2$	$0.0\pm 0.0$	$0.2\pm 0.1$	$0.0\pm 0.0$	$2.2\pm 1.0$	$0.6\pm 0.4$	$0.8\pm 0.5$	$0.0\pm 0.0$
Total misidentified	$30\pm 2$	$21\pm 1$	$9\pm 1$	$8\pm 1$	$229\pm 16$	$191\pm 14$	$93\pm 6$	$58\pm 4$
Wrong-sign prompt	–	$3.4\pm 0.4$	–	$1.9\pm 0.3$	–	$34\pm 4$	–	$10.3\pm 1.9$
Right-sign prompt	–	$6.5\pm 0.5$	–	$2.2\pm 0.1$	–	$35.4\pm 1.7$	–	$12.9\pm 0.3$
Total	-	$31\pm 1$	–	$12\pm 1$	–	$260\pm 14$	–	$81\pm 5$
Data	–	29	–	17	–	242	–	83

**Table 3:** Detailed breakdown of the symmetrised relative statistical, systematic and total uncertainties on the measurements of the $t\overline{t}$ production cross-section ${\sigma _{t\overline{t}}}$ at ${\sqrt{s}\,{=}\,7~\mathrm{TeV}}$ and ${\sqrt{s}=8~\mathrm{TeV}}$. Uncertainties quoted as ‘0.00’ are smaller than 0.005, whilst ‘–’ indicates the corresponding uncertainty is not applicable. The uncertainties on ${\epsilon _{e\mu }}$ and ${{C}_{b}}$ are also shown, with their relative signs indicated where relevant. They contribute with opposite signs to the uncertainties on ${\sigma _{t\overline{t}}}$, which also include uncertainties from estimates of the background terms ${N_1^\mathrm{bkg}}$ and ${N_2^\mathrm{bkg}}$. The lower part of the table gives the systematic uncertainties that are different for the measurement of the fiducial cross-section $\sigma _{t\overline{t}}^\mathrm{fid}$, together with the total analysis systematic and total uncertainties on $\sigma _{t\overline{t}}^\mathrm{fid}$

$\sqrt{s}$	7 TeV			8 TeV		
Uncertainty (inclusive ${\sigma _{t\overline{t}}}$)	$\varDelta {\epsilon _{e\mu }}/{\epsilon _{e\mu }}$ (%)	$\varDelta {{C}_{b}}/{{C}_{b}}$ (%)	$\varDelta {\sigma _{t\overline{t}}}/{\sigma _{t\overline{t}}}$ (%)	$\varDelta {\epsilon _{e\mu }}/{\epsilon _{e\mu }}$ (%)	$\varDelta {{C}_{b}}/{{C}_{b}}$ (%)	$\varDelta {\sigma _{t\overline{t}}}/{\sigma _{t\overline{t}}}$ (%)
Data statistics			1.69			0.71
$t\overline{t}$ modelling	0.71	$-0.72$	1.43	0.65	$-0.57$	1.22
Parton distribution functions	1.03	–	1.04	1.12	–	1.13
QCD scale choice	0.30	–	0.30	0.30	–	0.30
Single-top modelling	–	–	0.34	–	–	0.42
Single-top/$t\overline{t}$ interference	–	–	0.22	–	–	0.15
Single-top $Wt$ cross-section	–	–	0.72	–	–	0.69
Diboson modelling	–	–	0.12	–	–	0.13
Diboson cross-sections	–	–	0.03	–	–	0.03
$Z$+jets extrapolation	–	–	0.05	–	–	0.02
Electron energy scale/resolution	0.19	$-0.00$	0.22	0.46	0.02	0.51
Electron identification	0.12	0.00	0.13	0.36	0.00	0.41
Muon momentum scale/resolution	0.12	0.00	0.14	0.01	0.01	0.02
Muon identification	0.27	0.00	0.30	0.38	0.00	0.42
Lepton isolation	0.74	–	0.74	0.37	–	0.37
Lepton trigger	0.15	$-0.02$	0.19	0.15	0.00	0.16
Jet energy scale	0.22	0.06	0.27	0.47	0.07	0.52
Jet energy resolution	$-0.16$	0.08	0.30	$-0.36$	0.05	0.51
Jet reconstruction/vertex fraction	0.00	0.00	0.06	0.01	0.01	0.03
$b$-tagging	–	0.18	0.41	–	0.14	0.40
Misidentified leptons	–	–	0.41	–	–	0.34
Analysis systematics (${\sigma _{t\overline{t}}}$)	1.56	0.75	2.27	1.66	0.59	2.26
Integrated luminosity	–	–	1.98	–	–	3.10
LHC beam energy	–	–	1.79	–	–	1.72
Total uncertainty (${\sigma _{t\overline{t}}}$)	1.56	0.75	3.89	1.66	0.59	4.27

Uncertainty (fiducial $\sigma _{t\overline{t}}^\mathrm{fid}$)	$\varDelta {\epsilon _{e\mu }}/{\epsilon _{e\mu }}$ (%)	$\varDelta {{C}_{b}}/{{C}_{b}}$ (%)	$\varDelta$ $\sigma _{t\overline{t}}^\mathrm{fid}$/$\sigma _{t\overline{t}}^\mathrm{fid}$(%)	$\varDelta {\epsilon _{e\mu }}/{\epsilon _{e\mu }}$ (%)	$\varDelta {{C}_{b}}/{{C}_{b}}$ (%)	$\varDelta {\sigma _{t\overline{t}}}/{\sigma _{t\overline{t}}}$ (%)
$t\overline{t}$ modelling	0.84	$-0.72$	1.56	0.74	$-0.57$	1.31
Parton distribution functions	0.35	–	0.38	0.23	–	0.28
QCD scale choice	0.00	–	0.00	0.00	–	0.00
Other uncertainties (as above)	0.88	0.21	1.40	1.00	0.17	1.50
Analysis systematics ($\sigma _{t\overline{t}}^\mathrm{fid}$)	1.27	0.75	2.13	1.27	0.59	2.01
Total uncertainty ($\sigma _{t\overline{t}}^\mathrm{fid}$)	1.27	0.75	3.81	1.27	0.59	4.14

## Systematic uncertainties

The systematic uncertainties on the measured cross-sections ${\sigma _{t\overline{t}}}$ are shown in detail in Table 3 together with the individual uncertainties on ${\epsilon _{e\mu }}$ and ${{C}_{b}}$. A summary of the uncertainties on ${\sigma _{t\overline{t}}}$ is shown in Table 4. Each source of uncertainty was evaluated by repeatedly solving Eq. (1) with all relevant input parameters simultaneously changed by $\pm 1$ standard deviation. Systematic correlations between input parameters (in particular significant anti-correlations between ${\epsilon _{e\mu }}$ and ${{C}_{b}}$ which contribute with opposite signs to ${\sigma _{t\overline{t}}}$) were thus taken into account. The total uncertainties on ${\sigma _{t\overline{t}}}$ and ${\epsilon _{b}}$ were calculated by adding the effects of all the individual systematic components in quadrature, assuming them to be independent. The sources of systematic uncertainty are discussed in more detail below; unless otherwise stated, the same methodology was used for both $\sqrt{s}=7~$ TeV and $\sqrt{s}=8~$ TeV datasets.

- $t\overline{t}$ **modelling:** Uncertainties on ${\epsilon _{e\mu }}$ and ${{C}_{b}}$ due to the simulation of $t\overline{t}$ events were assessed by studying the predictions of different $t\overline{t}$ generators and hadronisation models as detailed in Sect. 3. The prediction for ${\epsilon _{e\mu }}$ was found to be particularly sensitive to the amount of hadronic activity near the leptons, which strongly affects the efficiency of the lepton isolation requirements described in Sect. 4. These isolation efficiencies were therefore measured directly from data, as discussed below. The remaining uncertainties on ${\epsilon _{e\mu }}$ relating to lepton reconstruction, identification and lepton–jet overlap removal, were evaluated from the differences between the predictions from the baseline Powheg + Pythia $t\overline{t}$ sample and a sample generated using MC@NLO + Herwig, thus varying both the hard-scattering event generator and the fragmentation and hadronisation model. The MC@NLO + Herwig sample gave a larger value of ${\epsilon _{e\mu }}$ but a smaller value of ${{C}_{b}}$. Additional comparisons of Powheg + Pythia samples with the AUET2 rather than P2011C tune and with Powheg + Herwig, i.e. changing only the fragmentation/hadronisation model, gave smaller uncertainties. The Alpgen + Herwig and Alpgen + Pythia samples gave values of ${\epsilon _{e\mu }}$ up to 2 % higher than that of Powheg+Pythia, due largely to a more central predicted $\eta$ distribution for the leptons. However, this sample uses a leading-order generator and PDFs, and gives an inferior description of the electron and muon $\eta$ distributions (see Fig. 3c, e), so was not used to set the systematic uncertainty on ${\epsilon _{e\mu }}$. In contrast, the Alpgen samples were considered in setting the uncertainty on ${{C}_{b}}$, taken as the largest difference between the predictions of Powheg + Pythia and any of the other generators. The effect of extra radiation in $t\overline{t}$ events was also considered explicitly by using pairs of simulation samples with different Pythia tunes whose parameters span the variations compatible with ATLAS studies of additional jet activity in $t\overline{t}$ events at ${\sqrt{s}\,{=}\,7~\mathrm{TeV}}$ [51], generated using both AcerMC + Pythia and Alpgen + Pythia. These samples predicted large variations in the lepton isolation efficiencies (which were instead measured from data), but residual variations in other lepton-related uncertainties and ${{C}_{b}}$ within the uncertainties set from other simulation samples.
- **Parton distribution functions:** The uncertainties on ${\epsilon _{e\mu }}$, ${{C}_{b}}$ and the $Wt$ single top background due to uncertainties on the proton PDFs were evaluated using the error sets of the CT10 NLO [11], MSTW 2008 68 % CL NLO [9, 10] and NNPDF 2.3 NLO [13] sets. The final uncertainty was calculated as half the envelope encompassing the predictions from all three PDF sets along with their associated uncertainties, following the PDF4LHC recommendations [8].
- **QCD scale choices:** The lepton $p_\mathrm{T}$ and $\eta$ distributions, and hence ${\epsilon _{e\mu }}$, are sensitive to the choices of QCD renormalisation and factorisation scales. This effect was investigated using $\sqrt{s}=8~$ TeV generator-level Powheg + Pythia $t\overline{t}$ samples where the two scales were separately varied up and down by a factor of two from their default values of $Q^2=$ ${m}_{t}$ $^2+p_{\mathrm{T},t}^2$. The systematic uncertainty for each scale was taken as half the difference in ${\epsilon _{e\mu }}$ values between the samples with increased and decreased QCD scale, and the uncertainties for the renormalisation and factorisation scales were then added linearly to give a total scale uncertainty of 0.30 % on ${\epsilon _{e\mu }}$, assumed to be valid for both centre-of-mass energies.
- **Single top modelling:** Uncertainties related to $Wt$ single top modelling were assessed by comparing the predictions from Powheg + Pythia, Powheg + Herwig, MC@NLO + Herwig, and AcerMC + Pythia with two tunes producing different amounts of additional radiation, in all cases normalising the total production rate to the approximate NNLO cross-section prediction. The resulting uncertainties are about 5 % and 20 % on the one and two $b$-tag background contributions. The background in the two $b$-tag sample is sensitive to the production of $Wt$ with an additional $b$-jet, a NLO contribution to $Wt$ which can interfere with the $t\overline{t}$ final state. The sensitivity to this interference was studied by comparing the predictions of Powheg with the diagram-removal (baseline) and diagram-subtraction schemes [33, 52], giving additional single-top/$t\overline{t}$ interference uncertainties of 1–2 % and 20 % for the one and two $b$-tag samples. The production of single top quarks in association with a $Z$ boson gives contributions which are negligible compared to the above uncertainties. Production of single top quarks via the $t$- and $s$-channels gives rise to final states with only one prompt lepton, and is accounted for as part of the misidentified-lepton background.
- **Background cross-sections:** The uncertainties on the $Wt$ single top cross-section were taken to be 7.6 % and 6.8 % at ${\sqrt{s}\,{=}\,7~\mathrm{TeV}}$ and ${\sqrt{s}=8~\mathrm{TeV}}$, based on Ref. [34]. The uncertainties on the diboson cross-sections were set to 5 % [36].
- **Diboson modelling:** Uncertainties in the backgrounds from dibosons with one or two additional $b$-tagged jets were assessed by comparing the baseline prediction from Alpgen + Herwig with that of Sherpa [40] including massive $b$ and $c$ quarks, and found to be about 20 %. The background from 125 GeV SM Higgs production in the gluon fusion, vector-boson fusion, and $WH$ and $ZH$ associated production modes, with $H\rightarrow WW$ and $H\rightarrow \tau \tau$, was evaluated to be smaller than the diboson modelling uncertainties, and was neglected.
- ***Z*** $+$ **jets extrapolation:** The uncertainties on the extrapolation of the $Z$+jets background from $Z\rightarrow ee/\mu \mu$ to $Z\rightarrow \tau \tau$ events result from statistical uncertainties, comparing the results from $ee$ and $\mu \mu$, which have different background compositions, and considering the dependence of the scale factors on $Z$ boson $p_\mathrm{T}$.
- **Lepton identification and measurement:** The modelling of the electron and muon identification efficiencies, energy scales and resolutions (including the effects of pileup) were studied using $Z\rightarrow ee/\mu \mu$, $J/\psi \rightarrow ee/\mu \mu$ and $W\rightarrow e\nu$ events in data and simulation, using the techniques described in Refs. [42, 43, 53]. Small corrections were applied to the simulation to better model the performance seen in data, and the associated systematic uncertainties were propagated to the cross-section measurement.
- **Lepton isolation:** The efficiency of the lepton isolation requirements was measured directly in data, from the fraction of selected opposite-sign $e\mu$ events with one or two $b$-tags where either the electron or muon fails the isolation cut. The results were corrected for the contamination from misidentified leptons, estimated using the same-sign $e\mu$ samples as described in Sect. 5, or by using the distributions of lepton impact parameter significance $|d_0|/\sigma _{d_0}$, where $d_0$ is the distance of closest approach of the lepton track to the event primary vertex in the transverse plane, and $\sigma _{d_0}$ its uncertainty. Consistent results were obtained from both methods, and showed that the baseline Powheg+Pythia simulation overestimates the efficiencies of the isolation requirements by about 0.5 % for both the electrons and muons. These corrections were applied to ${\epsilon _{e\mu }}$, with uncertainties dominated by the limited sizes of the same-sign and high impact-parameter significance samples used for background estimation. Similar results were found from studies in $Z\rightarrow ee$ and $Z\rightarrow \mu \mu$ events, after correcting the results for the larger average amount of hadronic activity near the leptons in $t\overline{t}\rightarrow e\mu \nu {\overline{\nu }}{b\overline{b}}$ events.
- **Jet-related uncertainties:** Although the efficiency to reconstruct and $b$-tag jets from $t\overline{t}$ events is extracted from the data, uncertainties in the jet energy scale, energy resolution and reconstruction efficiency affect the backgrounds estimated from simulation and the estimate of the tagging correlation ${{C}_{b}}$. They also have a small effect on ${\epsilon _{e\mu }}$ via the lepton–jet $\varDelta R$ separation cuts. The jet energy scale was varied in simulation according to the uncertainties derived from simulation and in-situ calibration measurements [47, 54], using a model with 21 (${\sqrt{s}\,{=}\,7~\mathrm{TeV}}$) or 22 (${\sqrt{s}=8~\mathrm{TeV}}$) separate orthogonal uncertainty components which were then added in quadrature. The jet energy resolution was found to be well modelled by simulation [55], and remaining uncertainties were assessed by applying additional smearing, which reduces ${\epsilon _{e\mu }}$. The calorimeter jet reconstruction efficiency was measured in data using track-based jets, and is also well described by the simulation; the impact of residual uncertainties was assessed by randomly discarding jets. The uncertainty associated with the jet vertex fraction requirement was assessed from studies of $Z\rightarrow ee/\mu \mu$+jets events.
- ***b*** **-tagging uncertainties:**   The efficiency for $b$-tagging jets from $t\overline{t}$ events was extracted from the data via Eq. (1), but simulation was used to predict the number of $b$-tagged jets and mistagged light-quark, gluon and charm jets in the $Wt$ single top and diboson backgrounds. The tagging correlation ${{C}_{b}}$ is also slightly sensitive to the efficiencies for tagging heavy- and light-flavour jets. The uncertainties in the simulation modelling of the $b$-tagging performance were assessed using studies of $b$-jets containing muons [50, 56], jets containing $D^{*+}$ mesons [57] and inclusive jet events [58].
- **Misidentified leptons:** The uncertainties on the number of events with misidentified leptons in the one and two $b$-tagged samples were derived from the statistical uncertainties on the numbers of same-sign lepton events, the systematic uncertainties on the opposite- to same-sign ratios $R_j$, and the uncertainties on the numbers of prompt same-sign events, as discussed in detail in Sect. 5.1. The overall uncertainties on the numbers of misidentified leptons vary from 30 to 50 %, dominated by the uncertainties on the ratios $R_j$.
- **Integrated luminosity:** The uncertainty on the integrated luminosity of the ${\sqrt{s}\,{=}\,7~\mathrm{TeV}}$ dataset is 1.8 % [59]. Using beam-separation scans performed in November 2012, the same methodology was applied to determine the ${\sqrt{s}=8~\mathrm{TeV}}$ luminosity scale, resulting in an uncertainty of 2.8 %. These uncertainties are dominated by effects specific to each dataset, and so are considered to be uncorrelated between the two centre-of-mass energies. The relative uncertainties on the cross-section measurements are slightly larger than those on the luminosity measurements because the $Wt$ single top and diboson backgrounds are evaluated from simulation, so are also sensitive to the assumed integrated luminosity.
- **LHC beam energy:** The LHC beam energy during the 2012 $pp$ run was calibrated to be $0.30\pm 0.66$ % smaller than the nominal value of 4 TeV per beam, using the revolution frequency difference of protons and lead ions during $p$+Pb runs in early 2013 [60]. Since this calibration is compatible with the nominal $\sqrt{s}$ of 8 TeV, no correction was applied to the measured ${\sigma _{t\overline{t}}}$ value. However, an uncertainty of 1.72 %, corresponding to the expected change in ${\sigma _{t\overline{t}}}$ for a 0.66 % change in $\sqrt{s}$ is quoted separately on the final result. This uncertainty was calculated using top++ 2.0, assuming that the relative change of ${\sigma _{t\overline{t}}}$ for a 0.66 % change in $\sqrt{s}$ is as predicted by the NNLLO+NNLL calculation. Following Ref. [60], the same relative uncertainty on the LHC beam energy is applied for the ${\sqrt{s}\,{=}\,7~\mathrm{TeV}}$ dataset, giving a slightly larger uncertainty of 1.79 % due to the steeper relative dependence of ${\sigma _{t\overline{t}}}$ on $\sqrt{s}$ in this region. These uncertainties are much larger than those corresponding to the very small dependence of ${\epsilon _{e\mu }}$ on $\sqrt{s}$, which changes by only 0.5 % between 7 and 8 TeV.
- **Top quark mass:** The simulation samples used in this analysis were generated with ${m}_{t}$ $=172.5$ GeV, but the acceptance for $t\overline{t}$ and $Wt$ events, and the $Wt$ background cross-section itself, depend on the assumed ${m}_{t}$ value. Alternative samples generated with ${m}_{t}$ varied in the range 165–180 GeV were used to quantify these effects. The acceptance and background effects partially cancel, and the final dependence of the result on the assumed ${m}_{t}$ value was determined to be ${\mathrm{d}{\sigma _{t\overline{t}}}/\mathrm{d}{{m}_{t}}}=-0.28~\%/\hbox {GeV}.$ The result of the analysis is reported assuming a fixed top mass of 172.5 GeV, and the small dependence of the cross-section on the assumed mass is not included as a systematic uncertainty.

**Table 4:** Summary of the relative statistical, systematic and total uncertainties on the measurements of the $t\overline{t}$ production cross-section ${\sigma _{t\overline{t}}}$ at ${\sqrt{s}\,{=}\,7~\mathrm{TeV}}$ and ${\sqrt{s}=8~\mathrm{TeV}}$

Uncertainty	$\varDelta {\sigma _{t\overline{t}}}/{\sigma _{t\overline{t}}}$ (%)	
$\sqrt{s}$	7 TeV	8 TeV
Data statistics	1.69	0.71
$t\overline{t}$ modelling and QCD scale	1.46	1.26
Parton distribution functions	1.04	1.13
Background modelling	0.83	0.83
Lepton efficiencies	0.87	0.88
Jets and $b$-tagging	0.58	0.82
Misidentified leptons	0.41	0.34
Analysis systematics (${\sigma _{t\overline{t}}}$)	2.27	2.26
Integrated luminosity	1.98	3.10
LHC beam energy	1.79	1.72
Total uncertainty	3.89	4.27

As shown in Tables 3 and 4, the largest systematic uncertainties on ${\sigma _{t\overline{t}}}$ come from $t\overline{t}$ modelling and PDFs, and knowledge of the integrated luminosities and LHC beam energy.

**Fig. 5:**
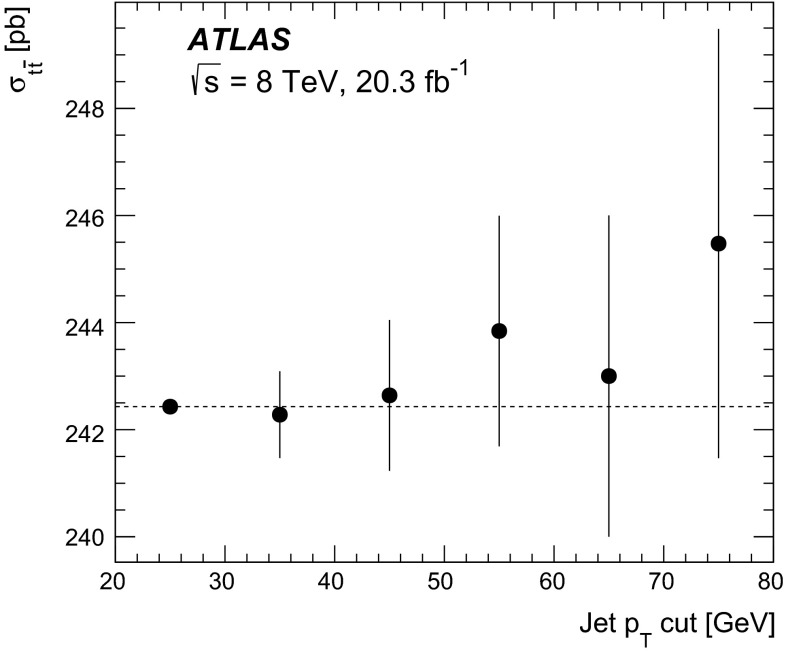
Measured $t\overline{t}$ cross-section at ${\sqrt{s}=8~\mathrm{TeV}}$ as a function of the $b$-tagged jet $p_\mathrm{T}$ cut. The *error bars* show the uncorrelated part of the statistical uncertainty with respect to the baseline measurement with jet $p_\mathrm{T}>25$ GeV

### Additional correlation studies

The tagging correlation ${{C}_{b}}$ was determined from simulation to be $1.009\pm 0.002\pm 0.007$ (${\sqrt{s}\,{=}\,7~\mathrm{TeV}}$) and $1.007\pm 0.002\pm 0.006$ (${\sqrt{s}=8~\mathrm{TeV}}$), where the first uncertainty is due to limited sizes of the simulated samples, and the second is dominated by the comparison of predictions from different $t\overline{t}$ generators. Additional studies were carried out to probe the modelling of possible sources of correlation. One possible source is the production of additional ${b\overline{b}}$ or ${c\overline{c}}$ pairs in $t\overline{t}$ production, which tends to increase both ${{C}_{b}}$ and the number of events with three or more $b$-tagged jets, which are not used in the measurement of ${\sigma _{t\overline{t}}}$. The ratio $R_{32}$ of events with at least three $b$-tagged jets to events with at least two $b$-tagged jets was used to quantify this extra heavy-flavour production in data. It was measured to be $R_{32}$ $=2.7\pm 0.4$ % (${\sqrt{s}\,{=}\,7~\mathrm{TeV}}$) and $2.8\pm 0.2$ % (${\sqrt{s}=8~\mathrm{TeV}}$), where the uncertainties are statistical. These values are close to the Powheg + Pythia prediction of $2.4\pm 0.1$ % (see Fig. 1), and well within the spread of $R_{32}$ values seen in the alternative simulation samples.

Kinematic correlations between the two $b$-jets produced in the $t\overline{t}$ decay could also produce a positive tagging correlation, as the efficiency to reconstruct and tag $b$-jets is not uniform as a function of $p_\mathrm{T}$ and $\eta$. For example, $t\overline{t}$ pairs produced with high invariant mass tend to give rise to two back-to-back collimated top quark decay systems where both $b$-jets have higher than average $p_\mathrm{T}$, and longitudinal boosts of the $t\overline{t}$ system along the beamline give rise to $\eta$ correlations between the two jets. These effects were probed by increasing the jet $p_\mathrm{T}$ cut in steps from the default of 25 GeV up to 75 GeV; above about 50 GeV, the simulation predicts strong positive correlations of up to ${{C}_{b}}\approx 1.2$ for a 75 GeV $p_\mathrm{T}$ cut. As shown for the $\sqrt{s}=8~$ TeV dataset in Fig. 5, the cross-sections fitted in data after taking these correlations into account remain stable across the full $p_\mathrm{T}$ cut range, suggesting that any such kinematic correlations are well modelled by the simulation. Similar results were seen at $\sqrt{s}=7~$ TeV. The results were also found to be stable within the uncorrelated components of the statistical and systematic uncertainties when tightening the jet and lepton $\eta$ cuts, raising the lepton $p_\mathrm{T}$ cut up to 55 GeV and changing the $b$-tagging working point between efficiencies of 60 % and 80 %. No additional uncertainties were assigned as a result of these studies.

## Results

Combining the estimates of ${\epsilon _{e\mu }}$ and ${{C}_{b}}$ from simulation samples, the estimates of the background ${N_1^\mathrm{bkg}}$ and ${N_2^\mathrm{bkg}}$ shown in Table 1 and the data integrated luminosities, the $t\overline{t}$ cross-section was determined by solving Eq. (1) to be:

$$\begin{aligned} {\sigma _{t\overline{t}}}&= 182.9\pm 3.1\pm 4.2\pm 3.6\pm 3.3~\mathrm{pb}~ ({\sqrt{s}\,{=}\,7~\mathrm{TeV}})\quad \mathrm{and} \\ {\sigma _{t\overline{t}}}&= 242.4\pm 1.7\pm 5.5\pm 7.5\pm 4.2~\mathrm{pb}~ ({\sqrt{s}=8~\mathrm{TeV}}), \end{aligned}$$

where the four uncertainties arise from data statistics, experimental and theoretical systematic effects related to the analysis, knowledge of the integrated luminosity and of the LHC beam energy. The total uncertainties are 7.1 pb (3.9 %) at $\sqrt{s}=7~$ TeV and 10.3 pb (4.3 %) at $\sqrt{s}=8~$ TeV. A detailed breakdown of the different components is given in Table 3. The results are reported for a fixed top quark mass of ${m}_{t}$ $=172.5$ GeV, and have a dependence on this assumed value of ${\mathrm{d}{\sigma _{t\overline{t}}}/\mathrm{d}{{m}_{t}}}=-0.28$ %/GeV. The product of jet reconstruction and $b$-tagging efficiencies ${\epsilon _{b}}$ was measured to be $0.557\pm 0.009$ at ${\sqrt{s}\,{=}\,7~\mathrm{TeV}}$ and $0.540\pm 0.006$ at ${\sqrt{s}=8~\mathrm{TeV}}$, in both cases consistent with the values in simulation.

**Fig. 6:**
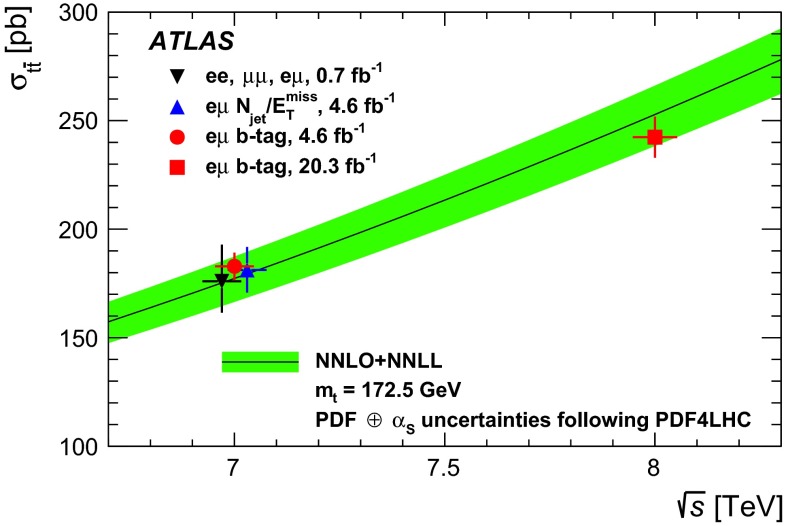
Measurements of the $t\overline{t}$ cross-section at ${\sqrt{s}\,{=}\,7~\mathrm{TeV}}$ and ${\sqrt{s}=8~\mathrm{TeV}}$ from this analysis ($e\mu$ $b$-tag) together with previous ATLAS results at ${\sqrt{s}\,{=}\,7~\mathrm{TeV}}$ using the $ee$, $\mu \mu$ and $e\mu$ channels [61] and using a fit to jet multiplicities and missing transverse momentum in the $e\mu$ channel [62]. The uncertainties in $\sqrt{s}$ due to the LHC beam energy uncertainty are displayed as *horizontal error bars*, and the *vertical error bars* do not include the corresponding cross-section uncertainties. The three ${\sqrt{s}\,{=}\,7~\mathrm{TeV}}$ measurements are displaced horizontally slightly for clarity. The NNLO+NNLL prediction [6, 7] described in Sect. 2 is also shown as a function of $\sqrt{s}$, for fixed ${m}_{t}$ $=172.5~{\hbox {GeV}}$ and with the uncertainties from PDFs, $\alpha _\mathrm{s}$ and QCD scale choices indicated by the *green band*

The results are shown graphically as a function of $\sqrt{s}$ in Fig. 6, together with previous ATLAS measurements of ${\sigma _{t\overline{t}}}$ at $\sqrt{s}=7~$ TeV in the $ee$, $\mu \mu$ and $e\mu$ dilepton channels using a count of the number of events with two leptons and at least two jets in an 0.7 ${\mathrm{fb}^{-1}}$ dataset [61], and using a fit of jet multiplicities and missing transverse momentum in the $e\mu$ dilepton channel alone with the full 4.6 ${\mathrm{fb}^{-1}}$ dataset [62]. The $\sqrt{s}=7~$ TeV results are all consistent, but cannot be combined as they are not based on independent datasets. The measurements from this analysis at both centre-of-mass energies are consistent with the NNLO+NNLL QCD calculations discussed in Sect. 2. The ${\sqrt{s}\,{=}\,7~\mathrm{TeV}}$ result is 13 % higher than a previous measurement by the CMS collaboration [63], whilst the ${\sqrt{s}=8~\mathrm{TeV}}$ result is consistent with that from CMS [64].

From the present analysis, the ratio of cross-sections ${R_{t\overline{t}}}={\sigma _{t\overline{t}}}$(8 TeV)/${\sigma _{t\overline{t}}}$(7 TeV) was determined to be:

$$\begin{aligned} {R_{t\overline{t}}}=1.326\pm 0.024 \pm 0.015 \pm 0.049 \pm 0.001 \end{aligned}$$

with uncertainties defined as above, adding in quadrature to a total of 0.056. The experimental systematic uncertainties (apart from the statistical components of the lepton isolation and misidentified lepton uncertainties, which were evaluated independently from data in each dataset) and the LHC beam energy uncertainty are correlated between the two centre-of-mass energies. The luminosity uncertainties were taken to be uncorrelated between energies. The result is consistent with the QCD NNLO+NNLL predicted ratio of $1.430\pm 0.013$ (see Sect. 2), which in addition to the quoted PDF, $\alpha _\mathrm{s}$ and QCD scale uncertainties varies by only $\pm 0.001$ for a $\pm 1$ GeV variation of ${m}_{t}$.

**Table 5:** Fiducial cross-section measurement results at ${\sqrt{s}\,{=}\,7~\mathrm{TeV}}$ and ${\sqrt{s}=8~\mathrm{TeV}}$, for different requirements on the minimum lepton $p_\mathrm{T}$ and maximum lepton $|\eta |$, and with or without the inclusion of leptons from $W\rightarrow \tau \rightarrow \ell$ decays. In each case, the first uncertainty is statistical, the second due to analysis systematic effects, the third due to the integrated luminosity and the fourth due to the LHC beam energy

$p^\ell _\mathrm{T}$ ( GeV)	$|\eta ^\ell |$	$W\rightarrow \tau \rightarrow \ell$	${\sqrt{s}\,{=}\,7~\mathrm{TeV}}$ (pb)	${\sqrt{s}=8~\mathrm{TeV}}$ (pb)
${>} 25$	${<}2.5$	Yes	$2.615\pm 0.044 \pm 0.056 \pm 0.052 \pm 0.047$	$3.448\pm 0.025 \pm 0.069 \pm 0.107 \pm 0.059$
${>} 25$	${<}2.5$	No	$2.305\pm 0.039 \pm 0.049 \pm 0.046 \pm 0.041$	$3.036\pm 0.022 \pm 0.061 \pm 0.094 \pm 0.052$
${>} 30$	${<}2.4$	Yes	$2.029\pm 0.034 \pm 0.043 \pm 0.040 \pm 0.036$	$2.662\pm 0.019 \pm 0.054 \pm 0.083 \pm 0.046$
${>} 30$	${<}2.4$	No	$1.817\pm 0.031 \pm 0.039 \pm 0.036 \pm 0.033$	$2.380\pm 0.017 \pm 0.048 \pm 0.074 \pm 0.041$

### Fiducial cross-sections

The preselection efficiency ${\epsilon _{e\mu }}$ can be written as the product of two terms ${\epsilon _{e\mu }}={{A}_{e\mu }}{{G}_{e\mu }}$, where the acceptance ${A}_{e\mu }$ represents the fraction of $t\overline{t}$ events which have a true opposite-sign $e\mu$ pair from $t\rightarrow W\rightarrow \ell$ decays (including via $W\rightarrow \tau \rightarrow \ell$), each with $p_\mathrm{T}>25$ GeV and within $|\eta |<2.5$, and ${G}_{e\mu }$ represents the reconstruction efficiency, i.e. the probability that the two leptons are reconstructed and pass all the identification and isolation requirements. A fiducial cross-section $\sigma _{t\overline{t}}^\mathrm{fid}$ can then be defined as ${\sigma _{t\overline{t}}^\mathrm{fid}}={{A}_{e\mu }}{\sigma _{t\overline{t}}}$, and measured by replacing ${\sigma _{t\overline{t}}}{\epsilon _{e\mu }}$ with ${\sigma _{t\overline{t}}^\mathrm{fid}}{{G}_{e\mu }}$ in Eq. (1), leaving the background terms unchanged. Measurement of the fiducial cross-section avoids the systematic uncertainties associated with ${A}_{e\mu }$, i.e. the extrapolation from the measured lepton phase space to the full phase space populated by inclusive $t\overline{t}$ production. In this analysis, these come mainly from knowledge of the PDFs and the QCD scale uncertainties. Since the analysis technique naturally corrects for the fraction of jets which are outside the kinematic acceptance through the fitted value of ${\epsilon _{b}}$, no restrictions on jet kinematics are imposed in the definition of $\sigma _{t\overline{t}}^\mathrm{fid}$. In calculating ${A}_{e\mu }$ and ${G}_{e\mu }$ from the various $t\overline{t}$ simulation samples, the lepton four-momenta were taken after final-state radiation, and including the four-momenta of any photons within a cone of size $\varDelta R=0.1$ around the lepton direction, excluding photons from hadron decays or produced in interactions with detector material. The values of ${A}_{e\mu }$ are about 1.4 % (including the $t\overline{t}\rightarrow e\mu \nu {\overline{\nu }}{b\overline{b}}$ branching ratio), and those of ${G}_{e\mu }$ about 55 %, at both centre-of-mass energies.

The measured fiducial cross-sections at $\sqrt{s}=7~$ TeV and $\sqrt{s}=8~$ TeV, for leptons with $p_\mathrm{T}>25$ GeV and $|\eta |<2.5$, are shown in the first row of Table 5. The relative uncertainties are shown in the lower part of Table 3; the PDF uncertainties are substantially reduced compared to the inclusive cross-section measurement, and the QCD scale uncertainties are reduced to a negligible level. The $t\overline{t}$ modelling uncertainties, evaluated from the difference between Powheg+Pythia and MC@NLO+Herwig samples increase slightly, though the differences are not significant given the sizes of the simulated samples. Overall, the analysis systematics on the fiducial cross-sections are 6–11 % smaller than those on the inclusive cross-section measurements.

Simulation studies predict that $11.9\pm 0.1$ % of $t\overline{t}$ events in the fiducial region have at least one lepton produced via $W\rightarrow \tau \rightarrow \ell$ decay. The second row in Table 5 shows the fiducial cross-section measurements scaled down to remove this contribution. The third and fourth rows show the measurements scaled to a different lepton fiducial acceptance of $p_\mathrm{T}>30$ GeV and $|\eta |<2.4$, a common phase space accessible to both the ATLAS and CMS experiments.

### Top quark mass determination

The strong dependence of the theoretical prediction for ${\sigma _{t\overline{t}}}$ on ${m}_{t}$ offers the possibility of interpreting measurements of ${\sigma _{t\overline{t}}}$ as measurements of ${m}_{t}$. The theoretical calculations use the pole mass ${m}_{t}^\mathrm{pole}$, corresponding to the definition of the mass of a free particle, whereas the top quark mass measured through direct reconstruction of the top decay products [65–68] may differ from the pole mass by $O(1$ GeV) [69, 70]. It is therefore interesting to compare the values of ${m}_{t}$ determined from the two approaches, as explored previously by the D0 [71, 72] and CMS [73] collaborations.

**Fig. 7:**
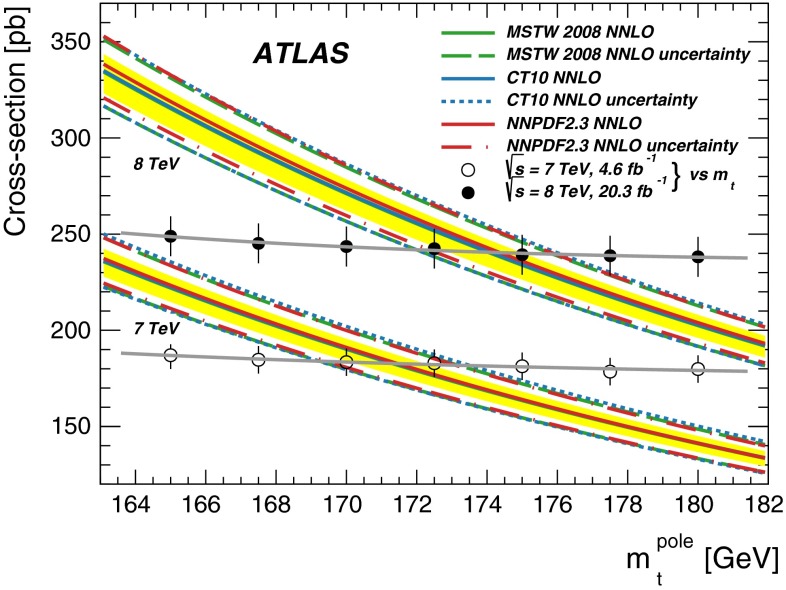
Predicted NNLO+NNLL $t\overline{t}$ production cross-sections at ${\sqrt{s}\,{=}\,7~\mathrm{TeV}}$ and ${\sqrt{s}=8~\mathrm{TeV}}$ as a function of ${m}_{t}^\mathrm{pole}$, showing the central values (*solid lines*) and total uncertainties (*dashed lines*) with several PDF sets. The *yellow band* shows the QCD scale uncertainty. The measurements of ${\sigma _{t\overline{t}}}$ are also shown, with their dependence on the assumed value of ${m}_{t}$ through acceptance and background corrections parameterised using Eq. (2)

The dependence of the cross-section predictions (calculated as described in Sect. 2) on ${m}_{t}^\mathrm{pole}$ is shown in Fig. 7 at both ${\sqrt{s}\,{=}\,7~\mathrm{TeV}}$ and ${\sqrt{s}=8~\mathrm{TeV}}$. The calculations were fitted to the parameterisation proposed in Ref. [6], namely:

2 $$\begin{aligned} {\sigma ^\mathrm{theo}_{t\overline{t}}}({{m}_{t}^\mathrm{pole}})=\sigma ({{m}_{t}^\mathrm{ref}})\left( \frac{{{m}_{t}^\mathrm{ref}}}{{{m}_{t}^\mathrm{pole}}}\right) ^4 (1+a_1x+a_2x^2) \end{aligned}$$

where the parameterisation constant ${{m}_{t}^\mathrm{ref}}=172.5$ GeV, $x=({{m}_{t}^\mathrm{pole}}-{{m}_{t}^\mathrm{ref}})/{{m}_{t}^\mathrm{ref}}$, and $\sigma ({{m}_{t}^\mathrm{ref}})$, $a_1$ and $a_2$ are free parameters. This function was used to parameterise the dependence of ${\sigma _{t\overline{t}}}$ on ${m}_{t}$ separately for each of the NNLO PDF sets CT10, MSTW and NNPDF2.3, together with their uncertainty envelopes.

Figure 7 also shows the small dependence of the experimental measurement of ${\sigma _{t\overline{t}}}$ on the assumed value of ${m}_{t}$, arising from variations in the acceptance and $Wt$ single top background, as discussed in Sect. 6. This dependence was also parameterised using Eq. (2), giving a derivative of ${\mathrm{d}{\sigma _{t\overline{t}}}/\mathrm{d}{{m}_{t}}}=-0.28\pm 0.03$ %/GeV at 172.5 GeV for both centre-of-mass energies, where the uncertainty is due to the limited size of the simulated samples. Here, ${m}_{t}$ represents the top quark mass used in the Monte Carlo generators, corresponding to that measured in direct reconstruction, rather than the pole mass. However, since this experimental dependence is small, differences between the two masses of up to 2 GeV have a negligible effect (${<}0.2$ GeV) on the pole mass determination. A comparison of the theoretical and experimental curves shown in Fig. 7 therefore allows an unambiguous extraction of the top quark pole mass.

The extraction is performed by maximising the following Bayesian likelihood as a function of the top quark pole mass ${m}_{t}^\mathrm{pole}$:

3 $$\begin{aligned} {\mathcal L}({{m}_{t}^\mathrm{pole}})&= \int G({\sigma ^{\prime }_{t\overline{t}}}|{\sigma _{t\overline{t}}}({{m}_{t}^\mathrm{pole}}),{\rho _\mathrm{exp}}) \nonumber \\&\quad \cdot G({\sigma ^{\prime }_{t\overline{t}}}|{\sigma ^\mathrm{theo}_{t\overline{t}}}({{m}_{t}^\mathrm{pole}}),\rho ^{\pm }_\mathrm{theo}~) \mathrm{d}{\sigma ^{\prime }_{t\overline{t}}}. \end{aligned}$$

Here, $G(x|\mu ,\rho )$ represents a Gaussian probability density in the variable $x$ with mean $\mu$ and standard deviation $\rho$. The first Gaussian term represents the experimental measurement ${\sigma _{t\overline{t}}}$ with its dependence on ${m}_{t}^\mathrm{pole}$ and uncertainty $\rho _\mathrm{exp}$. The second Gaussian term represents the theoretical prediction given by Eq. (2) with its asymmetric uncertainty $\rho ^{\pm }_\mathrm{theo}$ obtained from the quadrature sum of PDF+$\alpha _\mathrm{s}$ and QCD scale uncertainties evaluated as discussed in Sect. 2. The likelihood in Eq. (3) was maximised separately for each PDF set and centre-of-mass energy to give the ${m}_{t}^\mathrm{pole}$ values shown in Table 6. A breakdown of the contributions to the total uncertainties is given for the CT10 PDF results in Table 7; it can be seen that the theoretical contributions are larger than those from the experimental measurement of ${\sigma _{t\overline{t}}}$. A single ${m}_{t}^\mathrm{pole}$ value was derived for each centre-of-mass energy by defining an asymmetric Gaussian theoretical probability density in Eq. (3) with mean equal to the CT10 prediction, and a $\pm 1$ standard deviation uncertainty envelope which encompasses the $\pm 1$ standard deviation uncertainties from each PDF set following the PDF4LHC prescription [8], giving:

$$\begin{aligned} {{m}_{t}^\mathrm{pole}}&= 171.4\pm 2.6~{\hbox {GeV}}\ ({\sqrt{s}\,{=}\,7~\mathrm{TeV}})\quad \mathrm{and} \\ {{m}_{t}^\mathrm{pole}}&= 174.1\pm 2.6~{\hbox {GeV}}\ ({\sqrt{s}=8~\mathrm{TeV}}). \end{aligned}$$

Considering only uncorrelated experimental uncertainties, the two values are consistent at the level of 1.7 standard deviations. The top pole mass was also extracted using a frequentist approach, evaluating the likelihood for each ${m}_{t}^\mathrm{pole}$ value as the Gaussian compatibility between the theoretically predicted and experimentally measured values, and fixing the theory uncertainties to those at ${{m}_{t}^\mathrm{pole}}=172.5$ GeV. The results differ from those of the Bayesian approach by at most 0.2 GeV.

**Table 6:** Measurements of the top quark pole mass determined from the $t\overline{t}$  cross-section measurements at ${\sqrt{s}\,{=}\,7~\mathrm{TeV}}$ and ${\sqrt{s}=8~\mathrm{TeV}}$ using various PDF sets

PDF	${m}_{t}^\mathrm{pole}$( GeV) from ${\sigma _{t\overline{t}}}$	
	${\sqrt{s}\,{=}\,7~\mathrm{TeV}}$	${\sqrt{s}=8~\mathrm{TeV}}$
CT10 NNLO	$171.4\pm 2.6$	$174.1\pm 2.6$
MSTW 68 % NNLO	$171.2\pm 2.4$	$174.0\pm 2.5$
NNPDF2.3 5f FFN	$$ 171.3^{+2.2}_{-2.3}$$	$$ 174.2\pm 2.4$$

**Table 7:** Summary of experimental and theoretical uncertainty contributions to the top quark pole mass determination at ${\sqrt{s}\,{=}\,7~\mathrm{TeV}}$ and ${\sqrt{s}=8~\mathrm{TeV}}$ with the CT10 PDF set

$\varDelta {{m}_{t}^\mathrm{pole}}$ ( GeV)	${\sqrt{s}\,{=}\,7~\mathrm{TeV}}$	${\sqrt{s}=8~\mathrm{TeV}}$
Data statistics	0.6	0.3
Analysis systematics	0.8	0.9
Integrated luminosity	0.7	1.2
LHC beam energy	0.7	0.6
PDF+$\alpha _s$	1.8	1.7
QCD scale choice	$^{+0.9}_{-1.2}$	$^{+0.9}_{-1.3}$

**Fig. 8:**
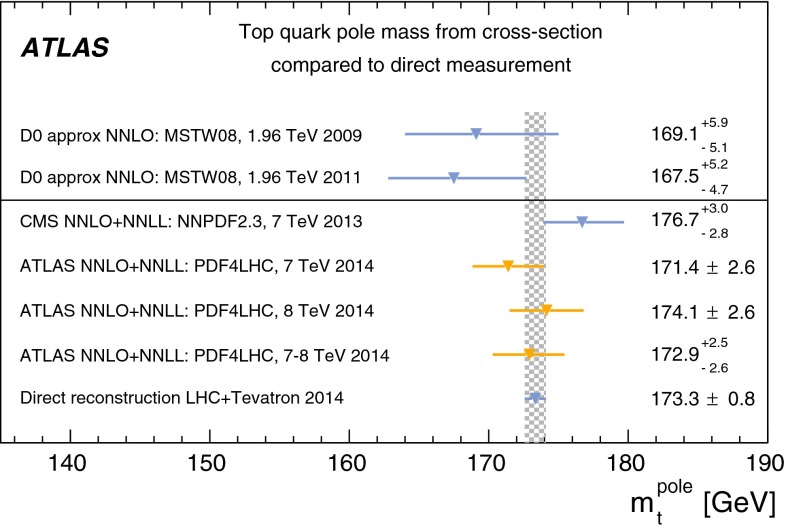
Comparison of top quark pole mass values determined from this and previous cross-section measurements [71–73]. The average of top mass measurements from direct reconstruction [74] is also shown

Finally, ${m}_{t}^\mathrm{pole}$ was extracted from the combined $\sqrt{s}=7~$ TeV and $\sqrt{s}=8~$ TeV dataset using the product of likelihoods (Eq. (3)) for each centre-of-mass energy and accounting for correlations via nuisance parameters. The same set of experimental uncertainties was considered correlated as for the cross-section ratio measurement, and the uncertainty on $\sigma ^\mathrm{theo}_{t\overline{t}}$ was considered fully correlated between the two datasets. The resulting value using the envelope of all three considered PDF sets is

$$\begin{aligned} {{m}_{t}^\mathrm{pole}}= 172.9^{+2.5}_{-2.6} ~{\hbox {GeV}}\end{aligned}$$

and has only a slightly smaller uncertainty than the individual results at each centre-of-mass energy, due to the large correlations, particularly for the theoretical predictions. The results are shown in Fig. 8, together with previous determinations using similar techniques from D0 [71, 72] and CMS [73]. All extracted values are consistent with the average of measurements from kinematic reconstruction of $t\overline{t}$ events of $173.34\pm 0.76$ GeV [74], showing good compatibility of top quark masses extracted using very different techniques and assumptions.

### Constraints on stop-pair production

Supersymmetry (SUSY) theories predict new bosonic partners for the Standard Model fermions and fermionic partners for the bosons. In the framework of a generic $R$-parity conserving minimal supersymmetric extension of the SM [75–79], SUSY particles are produced in pairs and the lightest supersymmetric particle is stable. If SUSY is realised in nature and responsible for the solution to the hierarchy problem, naturalness arguments suggest that the supersymmetric partners of the top quark—the top squarks or stops—should have mass close to ${m}_{t}$ in order to effectively cancel the top quark loop contributions to the Higgs mass [80, 81]. In this scenario, the lighter top squark mass eigenstate ${\tilde{t}_{1}}$ would be produced in pairs, and could decay via ${\tilde{t}_{1}}\rightarrow t{\tilde{\chi }_{1}^{0}}$ if ${m_{\tilde{t}_1}}>{{m}_{t}}+{m_{\tilde{\chi }_1^0}}$, where $\tilde{\chi }_{1}^{0}$, the lightest neutralino, is the lightest supersymmetric particle and is therefore stable. Stop-pair production could therefore give rise to $t\overline{t}{\tilde{\chi }_{1}^{0}}{\tilde{\chi }_{1}^{0}}$ intermediate states, appearing like $t\overline{t}$ production with additional missing transverse momentum carried away by the escaping neutralinos. The predicted cross-sections at $\sqrt{s}=8~$ TeV are about 40 pb for ${m_{\tilde{t}_1}}=175$ GeV, falling to 20 pb for 200 GeV. If the top squark mass $m_{\tilde{t}_1}$ is smaller than about 200 GeV, such events would look very similar to SM QCD $t\overline{t}$ production, making traditional searches exploiting kinematic differences very difficult, but producing a small excess in the measured $t\overline{t}$ cross-section, as discussed e.g. in Refs. [82, 83].

**Fig. 9:**
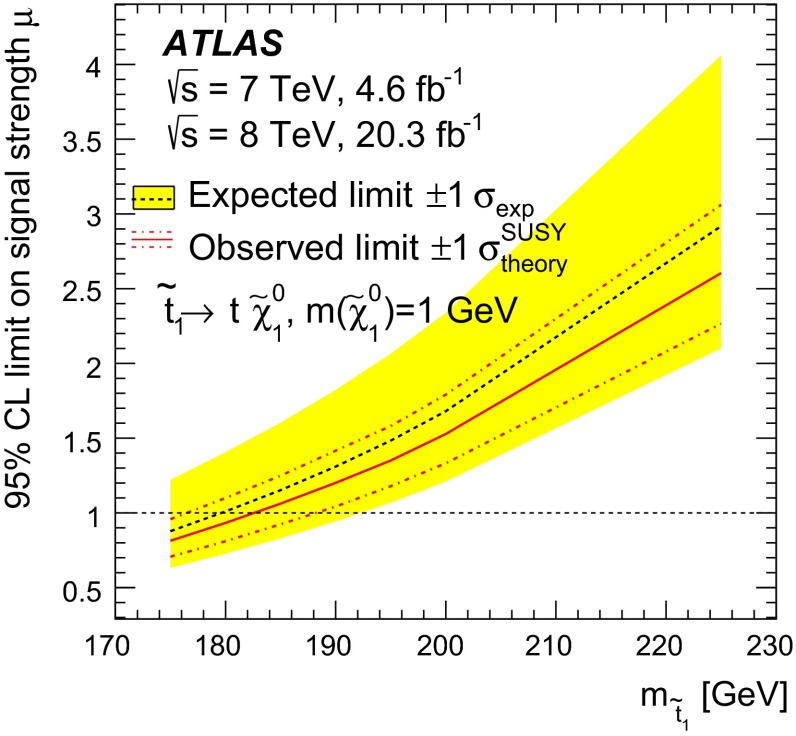
Expected and observed limits at 95 % CL on the signal strength $\mu$ as a function of $m_{\tilde{t}_1}$, for pair produced top squarks ${\tilde{t}_{1}}$ decaying with 100 % branching ratio via ${\tilde{t}_{1}}\rightarrow t{\tilde{\chi }_{1}^{0}}$ to predominantly right-handed top quarks, assuming ${m_{\tilde{\chi }_1^0}}=1$ GeV. The *black dotted line* shows the expected limit with $\pm 1\sigma$ contours, taking into account all uncertainties except the theoretical cross-section uncertainties on the signal. The *red solid line* shows the observed limit, with *dotted lines* indicating the changes as the nominal signal cross-section is scaled up and down by its theoretical uncertainty

The potential stop-pair signal yield was studied for top squark masses in the range 175–225 GeV and neutralino masses in the range 1 GeV$<{m_{\tilde{\chi }_1^0}}<{m_{\tilde{t}_1}}-{{m}_{t}}$ using simulated samples generated with Herwig++ [84] with the CTEQ6L1 PDFs [32], and NLO+NLL production cross-sections [85–87]. The mixing matrices for the top squarks and the neutralinos were chosen such that the top quark produced in the ${\tilde{t}_{1}}\rightarrow t{\tilde{\chi }_{1}^{0}}$ decay has a right-handed polarisation in 95 % of the decays. Due to the slightly more central $|\eta |$ distribution of the leptons from the subsequent $t\rightarrow Wq$, $W\rightarrow \ell \nu$ decay, the preselection efficiency ${\epsilon _{e\mu }}$ for these events is typically 10–20 % higher than for SM QCD $t\overline{t}$, increasing with $m_{\tilde{t}_1}$. However, the fraction of preselected events with one or two $b$-tagged jets is very similar to the SM case. The effect of a small admixture of stop-pair production in addition to the SM $t\overline{t}$ production is therefore to increase the measured cross-section by ${R_{\tilde{t}_1\tilde{t}_1}}{\sigma _{\tilde{t}_1\tilde{t}_1}}$, where $R_{\tilde{t}_1\tilde{t}_1}$is the ratio of ${\epsilon _{e\mu }}$ values for stop-pair and SM $t\overline{t}$ production, and $\sigma _{\tilde{t}_1\tilde{t}_1}$ is the stop-pair production cross-section.

Limits were set on stop-pair production by fitting the effective production cross-section ${R_{\tilde{t}_1\tilde{t}_1}}{\sigma _{\tilde{t}_1\tilde{t}_1}}$ multiplied by a signal strength $\mu$ to the difference between the measured cross-sections (${\sigma _{t\overline{t}}}$) and the theoretically predicted SM QCD production cross-sections ($\sigma ^\mathrm{theo}_{t\overline{t}}$). The two datasets were fitted simultaneously, assuming values of ${\sigma ^\mathrm{theo}_{t\overline{t}}}=177.3^{+11.5}_{-12.0}$ pb for $\sqrt{s}=7~$ TeV and $252.9^{+15.3}_{-16.3}$ pb for $\sqrt{s}=8~$ TeV, including the uncertainty due to a $\pm 1$ GeV variation in the top quark mass. The limits were determined using a profile likelihood ratio in the asymptotic limit [88], using nuisance parameters to account for correlated theoretical and experimental uncertainties.

The observed and expected limits on $\mu$ at the 95 % confidence level (CL) were extracted using the CLs prescription [89] and are shown in Fig. 9. Due to the rapidly decreasing stop-pair production cross-section with increasing $m_{\tilde{t}_1}$, the analysis is most sensitive below 180 GeV. Adopting the convention of reducing the estimated SUSY production cross-section by one standard deviation of its theoretical uncertainty (15 %, coming from PDFs and QCD scale uncertainties [90]), stop masses between the top mass threshold and 177 GeV are excluded, assuming 100 % branching ratio for ${\tilde{t}_{1}}\rightarrow t{\tilde{\chi }_{1}^{0}}$ and ${m_{\tilde{\chi }_1^0}}=1$ GeV. The limits from considering the ${\sqrt{s}\,{=}\,7~\mathrm{TeV}}$ and ${\sqrt{s}=8~\mathrm{TeV}}$ datasets separately are only slightly weaker, due to the large correlations in the systematic uncertainties between beam energies, particularly for the theoretical predictions. At each energy, they correspond to excluded stop-pair production cross-sections of 25–27 pb at 95 % CL.

The combined cross-section limits depend only slightly on the neutralino mass, becoming e.g. about 3 % weaker at ${m_{\tilde{t}_1}}=200$ GeV for ${m_{\tilde{\chi }_1^0}}=20$ GeV. However, they depend more strongly on the assumed top quark polarisation; in a scenario with ${m_{\tilde{t}_1}}=175$ GeV and ${m_{\tilde{\chi }_1^0}}=1$ GeV, and squark mixing matrices chosen such that the top quarks are produced with full left-handed polarisation, the limits are 4 % weaker than the predominantly right-handed case, rising to 14 % weaker for ${m_{\tilde{t}_1}}=200$ GeV. In this scenario, top squarks with masses from ${m}_{t}$ to 175 GeV can be excluded. Although the analysis has some sensitivity to three-body top squark decays of the form ${\tilde{t}_{1}}\rightarrow bW{\tilde{\chi }_{1}^{0}}$ for ${m_{\tilde{t}_1}}<{{m}_{t}}$, the $b$-jets become softer than those from SM $t\overline{t}$ production, affecting the determination of ${\epsilon _{b}}$. Therefore, no limits are set for scenarios with ${m_{\tilde{t}_1}}<{{m}_{t}}$.

## Conclusions

The inclusive $t\overline{t}$ production cross-section has been measured at the LHC using the full ATLAS 2011–2012 $pp$ collision data sample of 4.6 ${\mathrm{fb}^{-1}}$ at $\sqrt{s}=7~$ TeV and 20.3 ${\mathrm{fb}^{-1}}$ at $\sqrt{s}=8~$ TeV, in the dilepton $t\overline{t}\rightarrow e\mu \nu {\overline{\nu }}{b\overline{b}}$ decay channel. The numbers of opposite-sign $e\mu$ events with one and two $b$-tagged jets were counted, allowing a simultaneous determination of the $t\overline{t}$ cross-section ${\sigma _{t\overline{t}}}$ and the probability to reconstruct and $b$-tag a jet from a $t\overline{t}$ decay. Assuming a top quark mass of ${m}_{t}$ $=172.5$ GeV, the results are:

$$\begin{aligned} {\sigma _{t\overline{t}}}&= 182.9\,{\pm }\,3.1\,{\pm }\,4.2\,{\pm }\,3.6\,{\pm }\,3.3~\mathrm{pb}~ ({\sqrt{s}\,{=}\,7~\mathrm{TeV}})\quad \mathrm{and} \\ {\sigma _{t\overline{t}}}&= 242.4\,{\pm }\,1.7\,{\pm }\,5.5\,{\pm }\,7.5\,{\pm }\,4.2~\mathrm{pb}~ ({\sqrt{s}=8~\mathrm{TeV}}), \end{aligned}$$

where the four uncertainties arise from data statistics, experimental and theoretical systematic effects, knowledge of the integrated luminosity, and of the LHC beam energy, giving total uncertainties of 7.1 pb (3.9 %) and 10.3 pb (4.3 %) at $\sqrt{s}=7~$ TeV and $\sqrt{s}=8~$ TeV. The dependence of the results on the assumed value of ${m}_{t}$ is ${\mathrm{d}{\sigma _{t\overline{t}}}/\mathrm{d}{{m}_{t}}}=-0.28$ %/GeV, and the associated uncertainty is not included in the totals given above. The results are consistent with recent NNLO+NNLL QCD calculations, and have slightly smaller uncertainties than the theoretical predictions. The ratio of the two cross-sections, and measurements in fiducial ranges corresponding to the experimental acceptance, have also been reported.

The measured $t\overline{t}$ cross-sections have been used to determine the top quark pole mass via the dependence of the predicted cross-section on ${m}_{t}^\mathrm{pole}$, giving a value of ${{m}_{t}^\mathrm{pole}}=172.9^{+2.5}_{-2.6}$ GeV, compatible with the mass measured from kinematic reconstruction of $t\overline{t}$ events.

The results have also been used to search for pair-produced supersymmetric top squarks decaying to top quarks and light neutralinos. Assuming 100 % branching ratio for the decay ${\tilde{t}_{1}}\rightarrow t{\tilde{\chi }_{1}^{0}}$, and the production of predominantly right-handed top quarks, top squark masses between the top quark mass and 177 GeV are excluded at 95 % CL.

## Footnotes

1 Charge-conjugate modes are implied throughout.

2 ATLAS uses a right-handed coordinate system with its origin at the nominal interaction point in the centre of the detector, and the $z$ axis along the beam line. Pseudorapidity is defined in terms of the polar angle $\theta$ as $\eta =-\ln \tan {\theta /2}$, and transverse momentum and energy are defined relative to the beamline as $p_{t}=p\sin \theta$ and $E_\mathrm{T}=E\sin \theta$. The azimuthal angle around the beam line is denoted by $\phi$, and distances in $(\eta ,\phi )$ space by $\varDelta R=\sqrt{(\varDelta \eta ^2)+(\varDelta \phi )^2}$.
